# Tetratricopeptide repeat domain 7A is a nuclear factor that modulates transcription and chromatin structure

**DOI:** 10.1038/s41421-018-0061-y

**Published:** 2018-11-13

**Authors:** Marie-Therese El-Daher, Nicolas Cagnard, Marine Gil, Marie Chansel Da Cruz, Claire Leveau, Fernando Sepulveda, Mohammed Zarhrate, Frédéric Tores, Patricia Legoix, Sylvain Baulande, Jean Pierre de Villartay, Geneviève Almouzni, Jean-Pierre Quivy, Alain Fischer, Geneviève de Saint Basile

**Affiliations:** 1 Laboratory of Normal and Pathological Homeostasis of the Immune System, INSERM UMR 1163, Imagine Institute, Paris, France; 2 Université Paris Descartes -Sorbonne Paris Cité, Imagine Institute, Paris, France; 3 Bioinformatic Platform, INSERM UMR 1163, Université Paris Descartes -Sorbonne Paris Cité, Imagine Institute, Paris, France; 4 Structure Fédérative de Recherche (SFR) Necker, INSERM US24/CNRS UMS 3633, Paris, France; 5 Laboratory of Genome Dynamics in the Immune System, INSERM UMR1163, Paris, France; 6 Genomic Platform, INSERM UMR 1163, Imagine Institute, Paris, France; 7 ICGex Next-Generation Sequencing Platform, , Curie Institute, Paris, France; 8 CNRS UMR3664, Curie Institute, PSL Research University, Paris, France; 9 Université Sorbonne, Pierre et Marie Curie (UPMC), Paris, France; 10 Assistance Publique-Hôpitaux de Paris, Hôpital Necker-Enfants Malades Immunology and Pediatric Hematology Department, Paris, France; 11 Collège de France, Paris, France; 12 INSERM UMR1163, Paris, France; 13 Assistance Publique-Hôpitaux de Paris, Hôpital Necker-Enfants Malades, Centre d’Etudes des Déficits Immunitaires, Paris, France

## Abstract

A loss-of-function mutation in tetratricopeptide repeat domain 7A (TTC7A) is a recently identified cause of human intestinal and immune disorders. However, clues to related underlying molecular dysfunctions remain elusive. It is now shown based on the study of TTC7A-deficient and wild-type cells that TTC7A is an essential nuclear protein. It binds to chromatin, preferentially at actively transcribed regions. Its depletion results in broad range of epigenomic changes at proximal and distal transcriptional regulatory elements and in altered control of the transcriptional program. Loss of WT_TTC7A induces general decrease in chromatin compaction, unbalanced cellular distribution of histones, higher nucleosome accessibility to nuclease digestion along with genome instability, and reduced cell viability. Our observations characterize for the first time unreported functions for TTC7A in the nucleus that exert a critical role in chromatin organization and gene regulation to safeguard healthy immune and intestinal status.

## Introduction

The analysis of rare inherited diseases helps to identify molecular functions and events that are essential for human health. Recently, we and others reported that recessive loss-of-function mutations in the gene coding for tetratricopeptide repeat domain 7A (TTC7A) lead to immune and intestinal disorders of highly variable severity^1–8^. TTC7A deficiency is characterized by a progressive lymphopenia resulting in high susceptibility to a broad range of pathogens and minor or major intrinsic disruption of the digestive tract’s mucosal architecture extending from the stomach to the colon. The various consequences of TTC7A deficiency indicate that this protein is critical for fine-tuning of the balance between cells’ proliferation, differentiation, and survival. However, information on TTC7A’s cellular function(s) is still scarce. In vitro studies have shown that TTC7A deficiency causes inappropriate activation of RhoA-dependent effectors and thus disrupts cytoskeletal dynamics^1^. RhoA–ROCK targets are known to modulate the cytoskeletal assembly of actin, which has an important role in the regulation of cell contractility, motility, and morphology^9^. Accordingly, lymphocytes and gut epithelial cells from TTC7A-deficient patients exhibit impaired actin-related functions, such as increased spreading, adhesion, and cell polarity^1^. Moreover, TTC7A reportedly interacts with EFR3 homolog B and the phosphatidylinositol 4-kinase alpha, which is known to catalyze the production of phosphatidylinositol 4-phosphate on the plasma membrane in yeast and human cells^3,10^. This observation emphasizes the conservation, at least in part, of TTC7A’s functions from one species to another.

Natural mutants of TTC7A display partial or full impairments in protein expression. The tetratricopeptide repeats (TPRs) found in the TTC7A protein are predicted to form a platform that interacts with similar modules in other proteins or with unrelated sequence motifs^11^. TPR-containing proteins are involved in a variety of biological processes, including cell cycle regulation, transcriptional control, neurogenesis, and protein folding^12^. Interestingly, it was recently shown that TTC7B (the isoform of TTC7A) interacts with FAM126A, the absence of which leads to hypomyelinating leukoencephalopathy in humans^13^. Accordingly, the isoforms differ in their tissue distribution; TTC7A is highly expressed in hematopoietic and epithelial cells, whereas TTC7B is predominantly expressed in the brain and muscle (Database from BioGPS portal). Thus, TTC7A is likely to be involved in a wide range of protein complexes and hence functions.

In the present study, we further investigated TTC7A’s function at the subcellular level. We found that wild-type TTC7A (WT_TTC7A) was localized to several distinct cellular compartments including the nucleus and that this latter localization was severely affected when TTC7A was mutated. TTC7A associated to a chromatin, preferentially to actively transcribed regions. Its depletion resulted in a broad range of epigenomic changes at proximal and distal transcriptional regulatory elements and an altered control of the transcriptional program. Loss of WT_TTC7A induced unbalanced nucleosome assembly, a general decrease in chromatin compaction, increased in chromatin sensitivity to nuclease, genome instability, and reduced cell viability. Hence, we discovered a novel function of TTC7A linked to pathological states, an important modulator of both transcriptional activity and chromatin folding—both of which are mandatory to ensure productive response to various environmental stimuli and are crucial to maintain cell identity.

## Results

### TTC7A is a nuclear factor that is depleted upon loss-of-function mutations

**Fig. 1:**
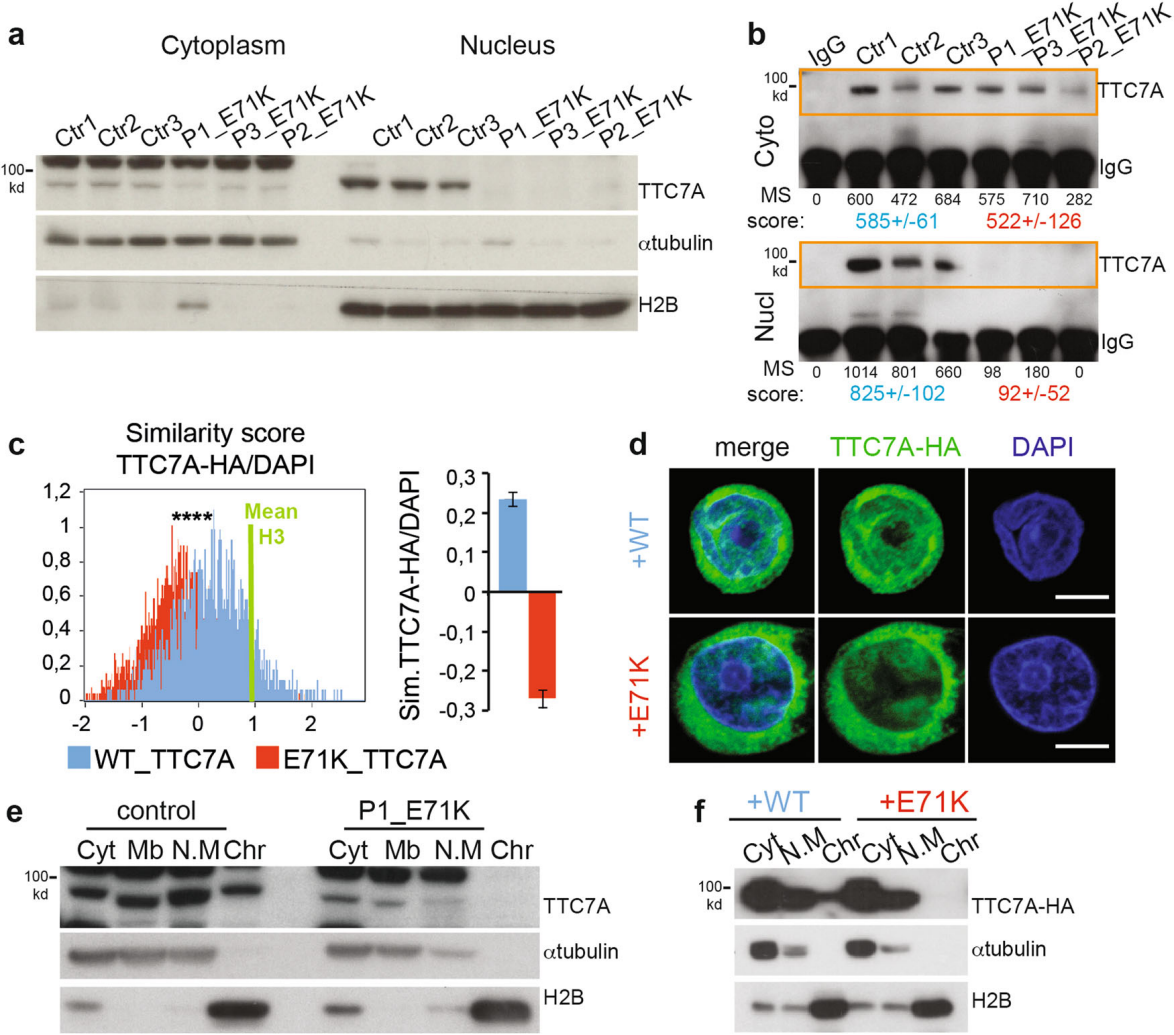
Mutant TTC7A is depleted from the nucleus and fails to bind with chromatin. **a** Subcellular fractionation of B lymphoblastoid cell lines (B-LCLs) from three controls and patients (P1-2-3_E71K). TTC7A (96 kDa) expression level was assessed in the cytoplasmic and nuclear fractions by western blot. α-tubulin and the total histone H2B were used to monitor the purity of the isolated fraction. **b** Immunoprecipitation of TTC7A protein in the cytoplasm (Cyto), and nuclear (Nucl) fraction using TTC7A or control IgG antibodies in three controls (Ctr) and three patients (P 1 to 3 _E71K) in B-LCLs. Immunoprecipitated fractions were resolved by western blot and sequenced by mass spectrometry. Abundance of TTC7A was scored after normalization. The mean score  ± SEM are indicated. **c** Cells expressing WT_ or E71K_TTC7A-HA were imaged on the ImageStream system and analyzed for HA-Alexa Fluor 488 and DAPI staining with IDEAS software. Correlation between WT (blue) and E71K_TTC7A (red) signals was measured using a similarity score. A mean similarity score for histone H3 staining and DAPI is provided as a positive control. Mean of three independent experiments, total number of cells in WT = 1723 and E71K = 2574, and unpaired t test *p*-value < 0.0001. **d** TTC7A cellular localization in B-LCLs by confocal microscopy. Cells expressing WT_ or E71K_TTC7A-HA were stained with HA (green) and DAPI (blue). Scale bar: 5 µm. **e** Isolated subcellular fractionation were subjected to western blot and assessed for TTC7A level and specific antibodies for each compartment. Cytoplasm (Cyt), membrane (Mb), nuclear matrix (N.M), and chromatin-bound (chr). **f** Control cells were transduced with WT or E71K TTC7A-HA-ires-GFP. Stably expressing cells were subjected to subcellular fractionation, similar to (**c**)

As a member of the TPR family, TTC7A is expected to mediate a wide range of interactions with proteins within several molecular complexes. In order to probe TTC7A’s cellular functions, we first assessed its cellular distribution in B-lymphoblastoid cell lines (B-LCLs) derived from both healthy donors and TTC7A-deficient patients. To do so, a fractionation procedure was used to separate the cytoplasm, membranes, nuclear matrix, and chromatin-bound proteins. In control cells, endogenous TTC7A was present in all four compartments and enriched in the nucleus (Supplementary Fig. S1a). In contrast, the overall expression of TTC7A was strongly reduced in patient cells. Depending on the mutation, the levels ranged from detectable at least in the cytoplasm fraction (for E71K_TTC7A) to almost undetectable in both fractions. Interestingly, the decrease of TTC7A amounts in mutant cells compared with the control was much more pronounced in the nucleus than in the cytosol/cytoplasm (Fig. 1a, Supplementary Fig. S1b). To further evaluate the loss of mutant TTC7A from the nuclei, we used an immunoprecipitation/mass spectrometry technique to quantify the amount of protein in B-LCLs. Under these conditions, control and patient (E71K) samples showed similar mean abundance of immunoprecipitated TTC7A in the cytoplasm (585 ± 61 vs. 522 ± 126, respectively). In contrast, the mean abundance of immunoprecipitated TTC7A from nuclear fractions was about ninefold lower in E71K_TTC7A cells than in the control cells (825 ± 102 vs. 92 ± 52, respectively) (Fig. 1b). Similarly, TTC7A was not detected in the nuclear fraction of primary T cells from E71K_ and A832*_TTC7A patients, whereas low levels were detected in the cytoplasm (Supplementary Fig. S1c, d). Hence, the nuclear localization observed for endogenous WT_TTC7A was lost for all natural TTC7A mutants that we screened. The proteins’ cellular localizations were further analyzed by single-cell image analysis using an ImageStream flow cytometer and microscopy. Given that TTC7A antibody does not work in immunofluorescence, we used cells expressing an HA epitope fused to TTC7A. We evaluated the amount of protein in the nucleus by measuring the pixel intensity correlation between TTC7A-HA-AF488 and DAPI images. DAPI positively correlated with WT_TTC7A levels and negatively correlated with E71K_TTC7A levels (Fig. 1c). Consistently, confocal microscopy experiments indicated a clear decrease of nuclear localization for E71K_TTC7A compared with WT_TTC7A (Fig. 1d). We next looked more in detail at the presence of mutant TTC7A on chromatin fraction. Consistent with above, we found that compared to WT_TTC7A, the mutant E71K_TTC7A severely decreased in the nucleoplasm fraction and most importantly was absent from the chromatin fraction (Fig. 1e). To validate that mutant TTC7A is depleted from chromatin, we overexpressed both WT_ and E71K_TTC7A fused to HA in B-LCLs. Although both constructs were expressed to a similar level, the mutant E71K_TTC7A was not detected in the chromatin fraction as compared with WT_TTC7A (Fig. 1f).

Taken as a whole, these results showed that WT TTC7A localizes in the cytoplasmic and various nuclear compartments and binds to chromatin, whereas mutant TTC7A does not accumulate in the nucleus and in the chromatin.

### TTC7A associates to chromatin

**Fig. 2:**
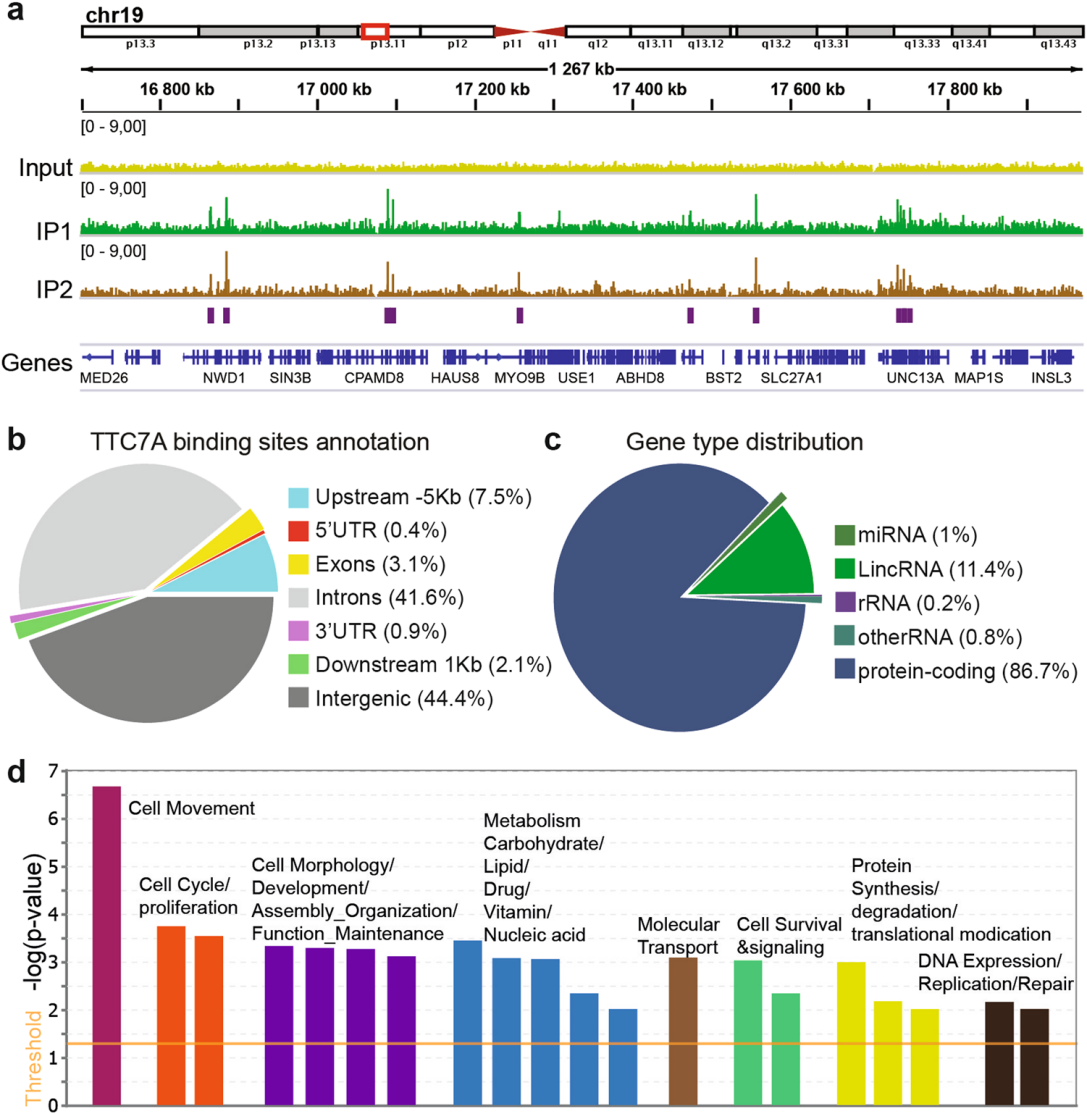
TTC7A is a nuclear factor that associates to chromatin. **a** TTC7A chromatin-binding sites are shown by using IGV genomic browser. Flag-tagged TTC7A ChIP-seq is presented as a pile-up of reads, and a representative enrichment on chromosome 19 is displayed. Bars depict significant enriched regions common in both IP1 and IP2 performed with two distinct antibodies. **b** ChIP-seq peaks annotation of TTC7A. **c** Gene type distribution of nearby genes for TTC7A peaks. TTC7A-enriched peaks are associated with the genes whose TSS is the closest and genes features category is provided. **d** Ingenuity pathways analysis (IPA) disease and function analysis. Genes that TSS localizes within 10 kb distance from the summit of TTC7A peaks (common IP1 and IP2 peaks) corresponded to functions such as cellular growth and maintenance, cell metabolism, survival, gene expression, and DNA damage

The nuclear detection of TTC7A prompted us to investigate whether TTC7A may have an essential role in this compartment that has not been yet considered. As TTC7A was present in the fraction of proteins bound to chromatin, we decided to build a genome-wide map of TTC7A’s interaction with chromatin by performing chromatin immunoprecipitation sequencing (ChIP-seq) experiment. Given that the specificity of TTC7A antibody in ChIP-seq was not validated, we used a lentiviral vector expressing Flag-tagged WT_TTC7A to create two control B-LCLs stably expressing Flag-TTC7A. First, subcellular fractionation showed that Flag-TTC7A expression was 12.5-fold higher than the endogenous level in the nucleoplasm and 3.5-fold higher in the chromatin-bound fraction (Supplementary Fig. 2a). We then used two Flag antibodies (hereafter referred to as IP1 and IP2) to perform TTC7A ChIP-seq. The results of these two independent ChIP experiments revealed a couple of thousands of Flag-TTC7A binding sites (Fig. 2a, Supplementary Table S1). Of note, since Flag-TTC7A expression on chromatin was 3.5-fold higher than the endogenous level, this may result in detecting non-specific binding that would not be observed under normal level of WT-TTC7A expression. The genomic annotation of TTC7A showed its predominant localization in introns and intergenic regions, as well as at proximal regulatory regions of genes (Fig. 2b, Supplementary Fig. S2b). DNA regions, nearby TTC7A binding peaks, were mostly protein coding on one hand and loci of long intergenic noncoding RNAs on the other hand (Fig. 2c, Supplementary Fig. S2c). To examine the relationship between TTC7A binding and its genomic environment, we focused on a set of high-confidence overlapping TTC7A binding peaks from IP1 and IP2 (*n* = 1177). We considered the genes localized within 10 kb up and downstream of these peaks and found that TTC7A-nearby-genes were involved in cellular functions such as cell division, organization and survival, metabolism and protein homeostasis, gene expression, and repair mechanisms (Fig. 2d). Moreover, analysis of TTC7A binding sequences motif pointed to two motifs that matched EGR1 and HOXA2 known consensus motif (Supplementary Fig. S2d). Both proteins are transcriptional regulator of genes required for differentiation and mitogenesis. These results suggest that TTC7A could be involved in key cellular functions required during cellular homeostasis and fate determination.

Together, these findings indicate that TTC7A associates with intergenic chromatin regions nearby protein-coding genes and thus might have a function in genomic expression.

### TTC7A is required for maintenance of the transcriptional program

**Fig. 3:**
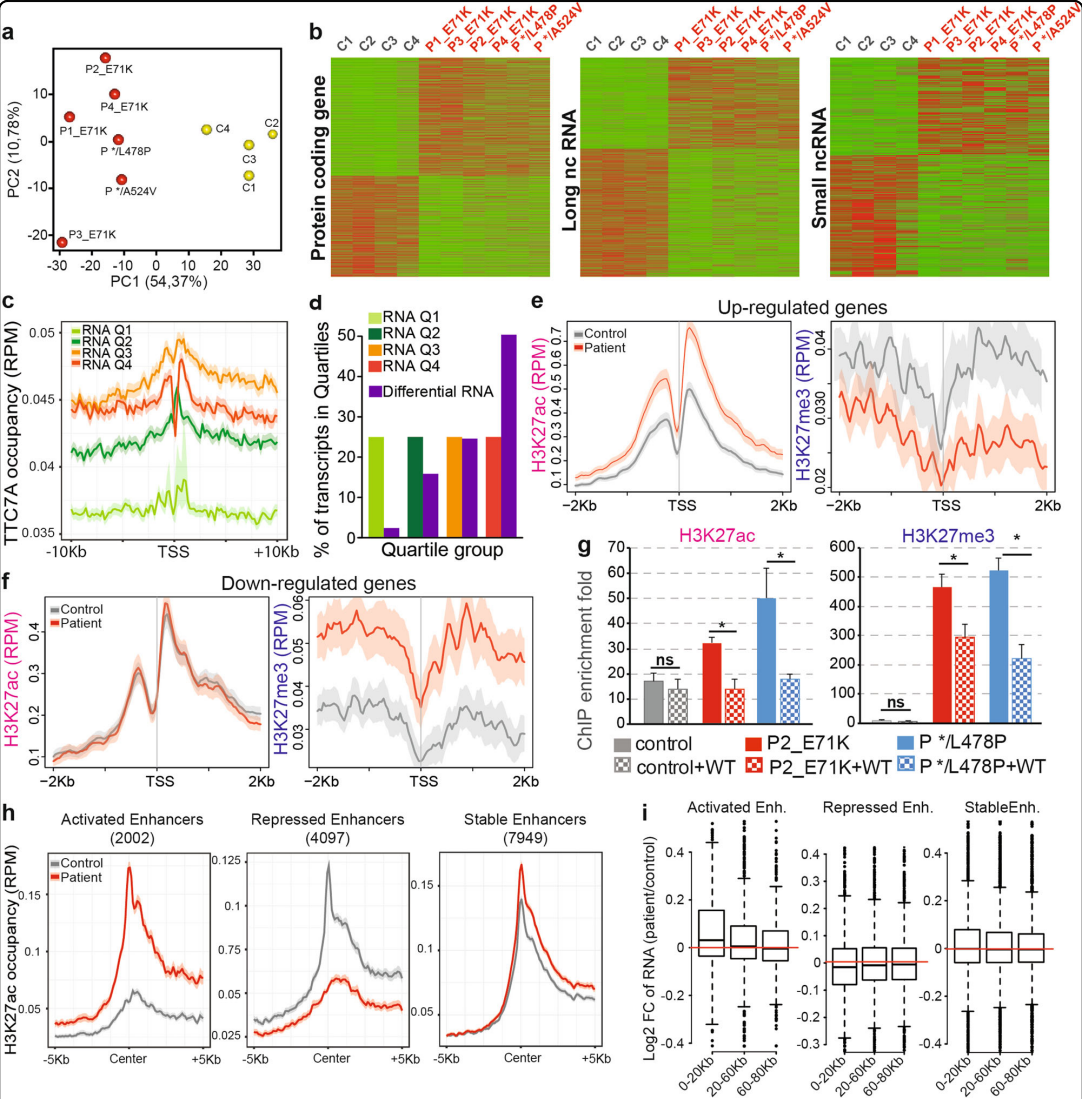
TTC7A deficiency deregulates the transcriptional activity of promoters and enhancers of active genes. **a** Principal component analysis (PCA) representing the variation between transcription profile of four controls (C1 to C4) and six patients (P1 to 4_E71K, P */L478P, and P */A524V). **b** Heatmaps for differently expressed transcripts in the four controls and six patients’ B lymphoblastoid cell lines (same as A). Transcription level was determined by HTA-2 microarray. Scale is relative to the population average (green: low; red: high) and *p* < 0.05; Student's *t* test. **c** Metaplots of TTC7A occupancy in control cells for all transcripts classified according to the expression in four groups containing the same number of transcripts, from lowest quartile 1 (Q1) to highest quartile 4 (Q4). RPM: reads per million mapped reads. **d** Proportion of differentially expressed transcripts in TTC7A-deficent cells (fold change > 1.2 and *p*-value < 0.05 between control and patients’ cells) among overall transcripts classified by quartiles. **e** and **f** Average profile plots of H3K27ac or H3K27me3 epigenetic markers at TSS regions of differentially expressed transcripts. Normalized level of H3k27ac and H3k27me3 in control and patients is depicted for up- and downregulated set of genes, **e** and **f**, respectively. **g** ChIP-quantitative PCR showing the amount of H3k27ac and H3k27me3 in two regions abnormally increased in patient cells. Left: a region highly acetylated, and right: a region highly methylated in patient cells. PCR quantification was obtained using a standard curve from the input DNA material. The fold enrichment was determined relatively to the level of control immunoglobulin ChIP. One control and two patients (P2_E71K and P */L478P) were assessed in non transduced and Flag-tagged WT_TTC7A transduced cells. Mean of two independent experiments ± SEM in duplicate. Unpaired *t* test; *p-value < 0.05. **h** Metaplots of H3K27ac occupancy at active, repressed, and unchanged enhancers in patient cells. Enhancers regions were set to be 2 kb upstream of TSS (transcription starting sites) and 10 kb downstream of TTS (transcription termination sites). **i** Box plots showing log2-fold change (FC) of transcripts level of genes 0–20, 20–60, and 60–80 kb nearby active, repressed, and unchanged enhancers in patient cells

We next examined the cellular impact of the loss of nuclear TTC7A in mutated cells. We first evaluated whether loss of nuclear TTC7A in patients’ cells alters the transcriptional program. Using HTA-2 microarrays, we established the overall transcriptional profile in control and patient cells. Principal component analysis of the gene expression data placed the control and mutant B-LCLs in different clusters. PC1 and PC2, respectively, accounted for 54% and 11% of the variations in the expression levels (Fig. 3a). Transcriptional changes included the up- and downregulated genes at a similar extent. Up- and downregulated transcripts belonged to various classes. Indeed, by applying a cutoff of *p* < 0.05, the differentially expressed transcripts corresponded to protein-coding transcripts (*n* = ~ 1000, of which 400 have a known function), long non-coding transcripts (*n* = ~ 900), and small non-coding RNAs (*n* = ~ 300) (Fig. 3b). Although some individual variations were noticed, control and patient cells differed significantly with regard to the mean transcript expression levels. A gene set enrichment analysis (GSEA) of differentially expressed genes revealed the enrichment of particular sets of genes that were predictive of (i) lymphocytes activation and death (upregulation of tumor necrosis factor alpha (TNFα) signaling via the NF-kB pathway), (ii) inflammatory responses and genes mediating caspase-dependent programmed cell death, and (iii) metabolic alteration including downregulation of hypoxia response, glycolysis, and mTORC1 complex activation (Supplementary Fig. S3a, b). These observations show that TTC7A deficiency lead to a broad pattern of changes with both up- and downregulated gene expression and thus indicate that a wide range of transcriptional perturbations occur in TTC7A-deficient cells.

We next used mass spectrometry to evaluate the impact of the altered transcription landscape on protein expression in TTC7A-deficient as compared with the control cells. Of the 4000 quantified proteins, 200 showed statistically significant variations in patient-derived cells that mostly consisted in upregulation (Supplementary Fig. S3c). These proteins are involved in several pathways, mainly RNA editing and gene expression (Supplementary Fig. S3d). A reliable correlation between the transcriptomic and proteomic data was found for about 75% of the differentially expressed genes. This finding strengthens our results obtained by combining the proteomic and genomic approaches, but also emphasizes the overall consistency of the differential expression profiles between WT and TTC7A mutated cells, thus supporting the hypothesis whereby TTC7A deficiency results in an altered control of the transcriptional program.

### TTC7A preferentially associates actively transcribed regions

In an attempt to link TTC7A genomic localization and changes in transcriptional regulation, we further probed the correlation between TTC7A enrichment and gene expression. Based on the expression level, transcripts were divided into four categories, from low to high. The occupancy of TTC7A at TSS (transcription starting site) was found higher for the group of genes belonging to the quartiles 3 and 4 of expression, as compared with the group of genes with lower expression level, namely quartiles 1 and 2 (Fig. 3c). This indicates that TTC7A binds the preferentially nearby transcriptional active genes. This is in line with the fact that the distribution of TTC7A regions over the genome is not random, suggesting binding specificities (Supplementary Fig. S4a, left). Indeed, TTC7A peaks were enriched in chromosomes containing strongly expressed genes (corresponding to the fourth expression quartile, Q4), with the exception of chromosome 12 (Supplementary Fig. S4a, middle), while being less frequently associated with chromosomes overall containing poorly expressed genes (corresponding to the first expression quartile, Q1) (Supplementary Fig. S4a, right). Interestingly, 75% of the differentially expressed genes in TTC7A-deficient cells corresponded to actively transcribed genes (expression quartiles Q3 and Q4) (Fig. 3d). We found that the up- and downregulated genes located within 10 kb of TTC7A-bound regions were involved with cellular functions matching the ones predicted from the list of differentially expressed genes, namely lymphocytes activation, cell death, and metabolism (Supplementary Figs. S3a, b, and S4b).

Together, these findings support the hypothesis whereby TTC7A deficiency results in transcriptional activity alteration of the active genes, partly localized nearby TTC7A binding sites.

### TTC7A deficiency modifies the epigenetic landscape and the activity of promoters and enhancers

Transcriptional regulation results from a combination of many nuclear processes, among which are histone post-translational modifications (PTM). In order to investigate whether histone PTM could be involved in the nuclear changes associated with TTC7A deficiency, we performed chromatin ChIP-seq of the H3K27ac and H3K27me3 histone marks that are associated, respectively, with transcriptional activation and repression (Supplementary Table S1). Control and patient cells did not differ significantly with regard to the overall coverage of H3K27ac and H3K27me3 (Supplementary Fig. S5a). In order to eliminate the inter-sample variability, we focused our analyses on the H3k27ac- or H3k27me3-associated regions that were reproducibly detected in the control cells on one hand (Cn) and patient cells on the other (Pn) (Supplementary Fig. S5b). As expected, proximal regulatory regions ( ± 2 kb TSSs) were marked by H3K27ac or H3K27me3 in both control and patient cells (Supplementary Fig. S5c). In patient cells, upregulated genes were associated with an increase in acetylation and a decrease in methylation (Fig. 3e). Conversely, downregulated genes were associated with an increase in methylation (Fig. 3f). Furthermore, the forced expression of Flag-tagged WT_TTC7A in patient cells (Supplementary Fig. S5d) completely restored H3K27ac and partially restored H3K27me3 to levels found in WT_TTC7A cells, as evaluated by ChIP-quantitative PCR assays of two genomic regions that we had previously identified as being affected (Fig. 3g). This indicates that epigenetic/histone PTMs alterations associated with transcriptional deregulation occurred at the promoter regions of TTC7A-deficient cells.

Global analysis revealed a distribution of TTC7A binding at both active promoter proximal regions and enhancers (Supplementary Fig. S5e). The occupancy of TTC7A at enhancers suggests that it could modulate the enhancer activity. To examine the relationship between the activation of enhancers and gene transcription in TTC7A-deficient cells, we first divided the enhancers into activated enhancers that exhibit an increase in H3K27ac mark, repressed enhancers that exhibit a decrease in H3K27ac mark, and stable enhancers that are unchanged for H3K27ac levels in patient cells (Fig. 3h). We then separated the genes into three groups according to their distance from the enhancers and observed that the strongest impact on gene expression level was observed for genes located within 20 kb from the enhancers (Fig. 3i, left). In contrast, gene expression pattern near or distant from the stable enhancers was relatively unchanged (Fig. 3i, right). A higher number of TTC7A binding sites were localized on repressed and stable enhancers as compared with activated enhancers (Supplementary Fig. S5f), suggesting an implication of TTC7A in the activation of a subset of enhancers.

Taken together, our data indicate that TTC7A deficiency introduces wide-ranging H3K27 tri-methylation and acetylation alterations at proximal and distal regulatory regions of the genome that are associated with major changes in the gene expression profile. Nonetheless, whether these epigenetic alterations were the consequence of a dysfunction in gene expression or conversely, the cause for the dysfunction of gene expression in TTC7A-deficient cells remains to be determined. Importantly, these PTMs alterations are not fixed, but are dynamic and TTC7A dependent, since in a mutant context they can be corrected by the expression of the Flag-tagged WT_TTC7A.

### TTC7A deficiency affects nuclear organization and chromatin compaction

**Fig. 4:**
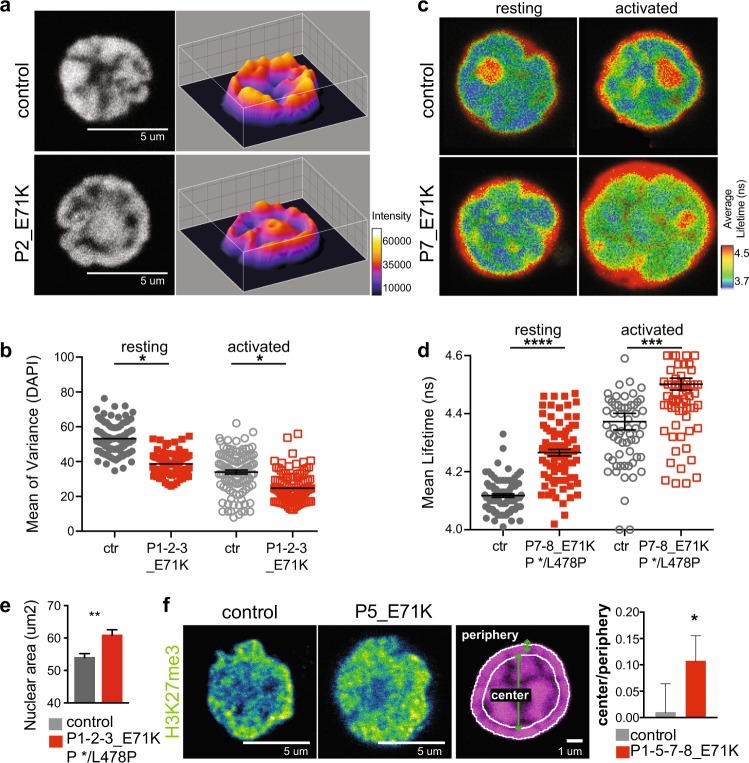
TTC7A deficiency modifies the structural organization of chromatin. **a** Left: confocal images of DAPI nuclear staining in control and patient purified naïve CD4+ T cells; scale bar: 5 µm. Right: corresponding interactive 3D surface plots illustrating the spatial intensity of DAPI. **b** DAPI intensity variation in control and patient CD4 T cells. Cells were isolated from peripheral blood, activated for 7 days in the culture, and analyzed for chromatin compaction. DAPI nuclear mask was applied and each nucleus was subdivided into squares of 12 pixels size in order to calculate the DAPI intensity variance. Mean of three independent experiments ± SEM. Total number of resting cells from controls (*n* = 100) and patients (*n* = 93); Total number of activated cells from controls (*n* = 109) and patients (*n* = 60). Turkey’s multiple comparisons test multiples *p*-value < 0.05. **c** Fluorescence lifetime maps in control and patient nuclei. Fluorescence excitation decay of YoYo-1 bound to DNA was measured by symphotime software. All four images are 9.59 µm width. **d** Mean fluorescence lifetime ± SEM of the whole nucleus was calculated in resting and activated CD4+ T cells. Mean of three independent experiments ±SEM. Total number of resting cells from controls (*n* = 88) and patients (*n* = 85). Total number of activated cells from controls (*n* = 77) and patients (*n* = 79). Turkey’s multiple comparisons test multiples; ****p-value < 0.0001; ***p-value < 0.001. **e** The nuclear area of the focal plane of control and patient B lymphoblastoid cell lines. Cells were stained with DAPI and imaged on the ImageStream system, then analyzed with IDEAS software. Area was measured using the feature mask-morphology (µm^2^). Graph is a mean ± SEM of three independent experiments with five independent controls and four patients. Total number of cells in controls = 27,191 and patients = 21,186. Unpaired *t* test *p*-value < 0.0077. **f** Confocal microscopy immunostainings of H3k27me3 (green fire blue). We quantified the intensity ratio (log10) of H3k27me3 between the center and the peripheral zone of activated T lymphocytes nuclei. The peripheral zone is set at 10 pixels from the outer layer of the nucleus; the rest is considered to be the center. Scale bar: 5 µm and 1 µm. Mean ± SEM of five independent experiments. Total number of cells in controls = 413 and patients = 500; Paired *t* test on means; *p-value < 0.0364

Higher-order chromatin structure contributes to the regulation of gene expression^14,15^. We therefore investigated whether a defect in the genome organization was associated with TTC7A deficiency. Firstly, we used microscopy imaging of DAPI staining to monitor the chromatin overall organizational state of the cell nucleus. This analysis revealed consistent alterations in activated primary T cells from patients as compared with the control cells. In control cells, the intensity of the DAPI staining was heterogeneous, with sharp transitions to dense areas that appeared to be individual domains. In contrast, the DAPI staining was more homogeneous and evenly distributed in E71K_ and */L478P_TTC7A cells, with a decrease of DAPI dense domains (Fig. 4a). These domains are usually associated with high levels of chromatin compaction, as found for instance in pericentric heterochromatin domains in mouse cells^16^. It is also well established that nuclear DNA is highly condensed in naive T cells, but opens up once the cells have been activated; this change is associated with gene expression^17^. We therefore assessed the texture of the DAPI staining pattern in purified naive CD4+ T cells in the resting state and following in vitro activation. To do so, we subdivided the nucleus images into equal-squared zones and quantified the variation of DAPI intensity (as determined by the variance). In control cells, we observed a decrease variation of intensity upon activation reflecting expected texture changes. In contrast, in E71K_TTC7A cells, the variation in intensity was reduced compared with the control cells in both resting and activated states (Fig. 4b), in line with a decreased condensation in the mutated TTC7A cells. Upon introduction of the Flag-tagged WT_TTC7A into activated CD4+ T lymphocytes cells, the variation of DAPI intensity was restored to control level. These results indicate that TTC7A is required to maintain proper chromatin distribution within the nucleus (Supplementary Fig. S6a).

In order to determine whether the modified DNA staining pattern in TTC7A mutated cells reflects a chromatin compaction defect rather than a change in organization, we next used fluorescence lifetime imaging (FLIM) to monitor the spatial condensation of the chromatin^18^. The DNA of fixed CD4+ T lymphocytes was stained with YoYo-1 fluorescent dye, then the mean fluorescence lifetime in control and patient nuclei was measured after excitation with a pulsed 471-nm laser (Fig. 4c). The fluorescence lifetime was found to be longer in both freshly isolated and in vitro-activated E71K_ and */L478P_TTC7A T lymphocytes than in control cells, indicating a lower compaction for the mutants in both resting and activated status (Fig. 4d). These observations are consistent with the DAPI pattern results and point to a loss of chromatin compaction/condensation in TTC7A-deficient cells. It is also known that the nucleus increases in size concomitantly with chromatin decondensation^19^. We therefore evaluated the surface area of the nucleus in patient cells and observed a mean increase (in the focal plan) of about 7 µm^2^ as compared with control area (Fig. 4e), in line with a decreased chromatin compaction. Taken together, these results indicate that the chromatin compaction and organization are abnormal in TTC7A-deficient cells, and suggest that TTC7A’s role in chromatin organization does not depend on the cell’s activation status.

Given that we found an alteration in the H3K27 tri-methylation and acetylation status (see above), we checked if the organization of those marks within the nucleus was also impacted. Hence, we determined the respective distribution of H3K27me3 and H3K27ac in global nuclei by immunofluorescence. In control cells, the H3K27me3 staining was concentrated at the nuclear periphery, in contrast to H3K27ac staining (Supplementary Fig. S6b). In TTC7A-deficient cells, an increased H3K27me3 localization was observed in the center of the nucleus at the expense of nuclear periphery, as shown by the higher H3K27me3 mark central/peripheral ratio (Fig. 4f, Supplementary Fig. S6c). The distribution in H3K27ac mark remained unchanged (Supplementary Fig. S6c). Thus, the absence of TTC7A impairs the topological distribution of the repressive histone mark H3K27me3 in the nuclei of mutant cells.

Collectively, these data indicate that TTC7A-deficient cells exhibit a decrease in chromatin compaction associated with an abnormal chromatin organization in the nucleus of interphasic cells, irrespective of the resting/activated state.

### TTC7A is required for histones dynamics and nucleosomal organization

**Fig. 5:**
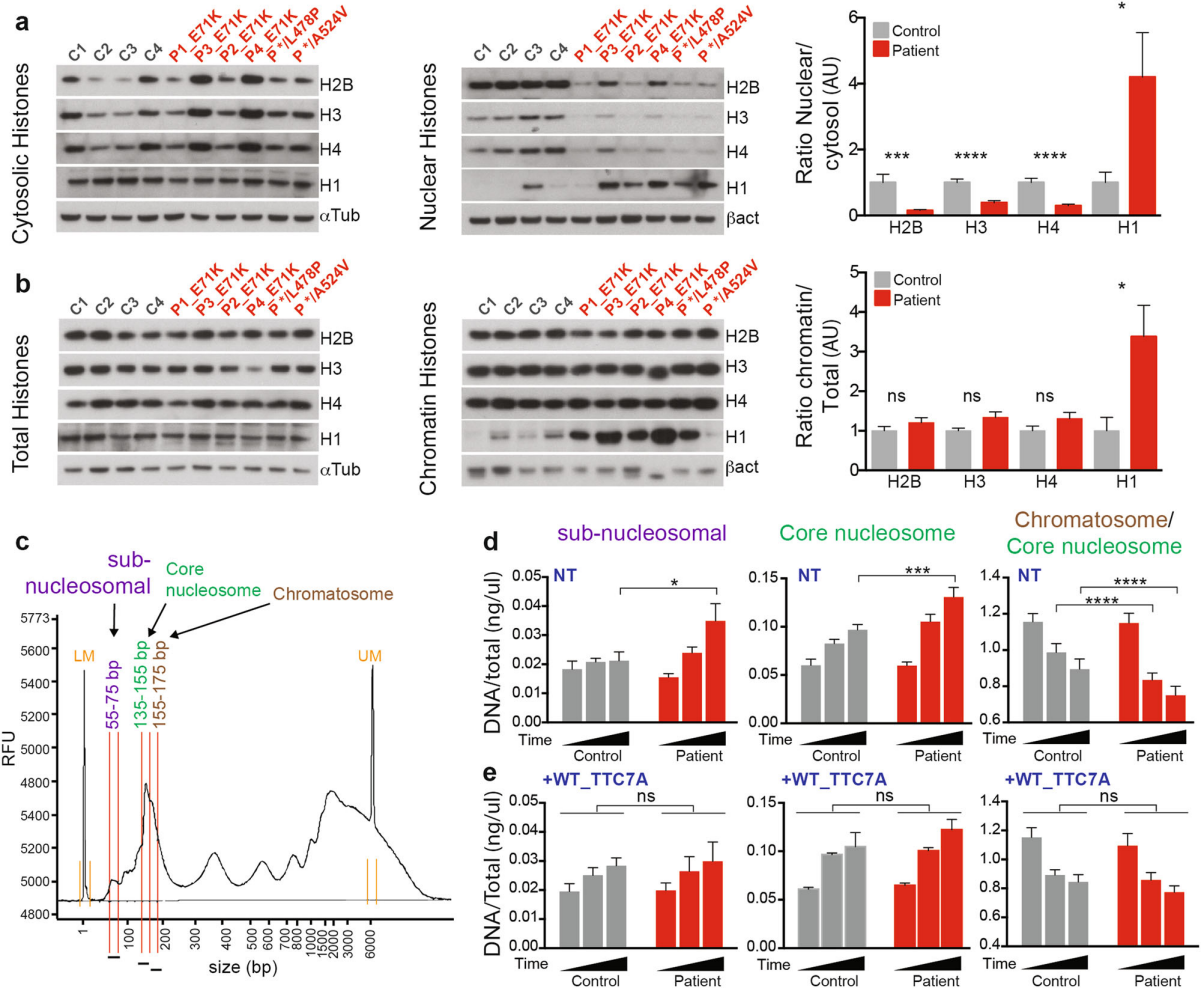
TTC7A is required for the proper histones distribution and chromatin packaging. **a** and **b** Histones subcellular fractionation in control and patient B lymphoblastoid cell lines. Histones H2B, H3, H4, and H1 were analyzed by western blot. Fractions are total histones, cytosolic histones (soluble pool), nuclear histones (salt-extractable pool), and chromatin histones (DNA enwrapped pool). The four controls are compared with six patient samples, and quantification is relative to control that is set to 1. The signal intensities of histones were normalized to internal loading control, α-tubulin (αtub), for total and cytosolic fractions; β-actin (βact) for nuclear and chromatin fractions. Graphs represent the ratio between nuclear vs. cytosolic (**a**) and chromatin-bound vs. total pools (**b**). Mean ± SEM of four independent experiments. Unpaired *t* test; *p-value < 0.05, ***p-value = 0.0003, ****p-value < 0.0001. **c** DNA lengths distribution upon micrococcal nuclease digestion of chromatin. Lengths of DNA fragments were measured and quantified using the fragment analyzer software. The scheme depicts the range of DNA sizes forming to subnucleosomal DNA (centered around 60 bp), forming the nucleosomal core particle (centered around 145 bp) and forming the chromatosome (centered around 165 bp). LM: lower marker, UM: upper marker. **d** A kinetic of micrococcal nuclease digestion of chromatin was conducted in control and patient B lymphoblastoid cell lines (P1_E71K, P2_E71K, and P */L478P). DNA concentration was measured and normalized to total DNA concentration. The mean of each time point was compared between control and patients. Mean ± SEM of six independent experiments. Sidak’s multiple comparisons test; *p-value < 0.05, ***p-value < 0.001, ****p-value < 0.0001. **e** Micrococcal nuclease digestion kinetics same as (**d**). Control and patient cells lines were stably expressing Flag-tagged WT_TTC7A. The mean of each time point was compared between control and patients. Mean ±  SEM of five independent experiments. Sidak’s multiple comparisons test; ns non-significant

To further investigate the decrease of chromatin compaction in TTC7A-deficient cells, we monitored the nucleosomal status of TTC7A-deficient cells; given the fact that chromatin compaction is achieved by nucleosomal organization. We first analyzed the amounts of histones and their distributions amongst cellular compartments. We determined the global amount of histones present in control and patient B-LCLs, and found no significant difference in total levels of histones H2B, H3, H4, and H1 (Supplementary Fig. S7a–d). However, it was found that the distribution of H2B, H3, and H4 between cytosolic and salt-extractable nuclear fractions was unbalanced when comparing control and patient cells. The ratios between nuclear and cytosolic H2B, H3, and H4 decreased in the absence of TTC7A. This observation indicates a trend for core histones to increase in the cytoplasm at the expense of the nuclear pool (Fig. 5a, Supplementary Fig. S7a–d). This alteration of histones distribution might impact the proper nucleosome assembly and disassembly equilibrium in TTC7A-deficient cells. Yet, the amount of H2B, H3, and H4 histones enwrapped by the chromatin did not differ between control and TTC7A-deficient cells (Fig. 5b, Supplementary Fig. S7a–d). Hence, although the core histones detected in the chromatin is not influenced by TTC7A expression, the efficacy of histones exchange or dynamics is modified when TTC7A is defective in the nucleus.

Interestingly, it was observed that, in TTC7A-deficient cells the expression level of the linker histone H1 was three to fourfold higher, both in the nuclear and chromatin pools, relatively to cytosolic and total pools, respectively, as compared with WT_TTC7A cells (Fig. 5a, b, Supplementary Fig. S7a–d). Due to the role of linker histone H1 in the structural and functional settings of chromatin organization^20–22^, this led us to analyze in more detail the DNA accessibility and nucleosomal organization by using a micrococcal nuclease (MNase) digestion assay. Such assay reveals DNA fragments of decreasing sizes. These fragments include the chromatosome 155-175 bp (core+linker histones), the nucleosome core particle 135-155 bp (core histones only) and DNA fragments of subnucleosomal size 55-75 bp (Fig. 5c)^23^. These fragments correspond to DNA protected from digestion by transcription factors^24,25^. In TTC7A-deficient cells, we observed a higher accumulation of subnucleosomal 55–75 bp DNA over time (Fig. 5d, left) and an increase of core nucleosomal DNA with a decrease of chromatosome relative to controls (Fig. 5d, middle and right). These results indicate that in TTC7A-deficient cells, the DNA accessibility and nucleosomal organization are altered compared with the control cells. This is consistent with the analysis by microscopy of nuclei from TTC7A-deficient cells that showed a decrease of chromatin compaction (Fig. 4). Most importantly, the introduction of Flag-tagged WT_TTC7A into mutant cells restored the level of subnucleosomal, nucleosomal cores and chromatosomes DNA fragments to those of normal control cells, indicating a specific role of TTC7A (Fig. 5e).

Taken as a whole, these findings indicate that TTC7A is essential to maintain the appropriate distribution of histones pools and the elementary organization of nucleosomes, which in the context of TTC7A deficiency likely impacts the chromatin compaction, higher-order chromatin structures, and ultimately, nuclear organization in interphasic cells.

### TTC7A-deficient cells fail to fold the mitotic chromosomes properly

**Fig. 6:**
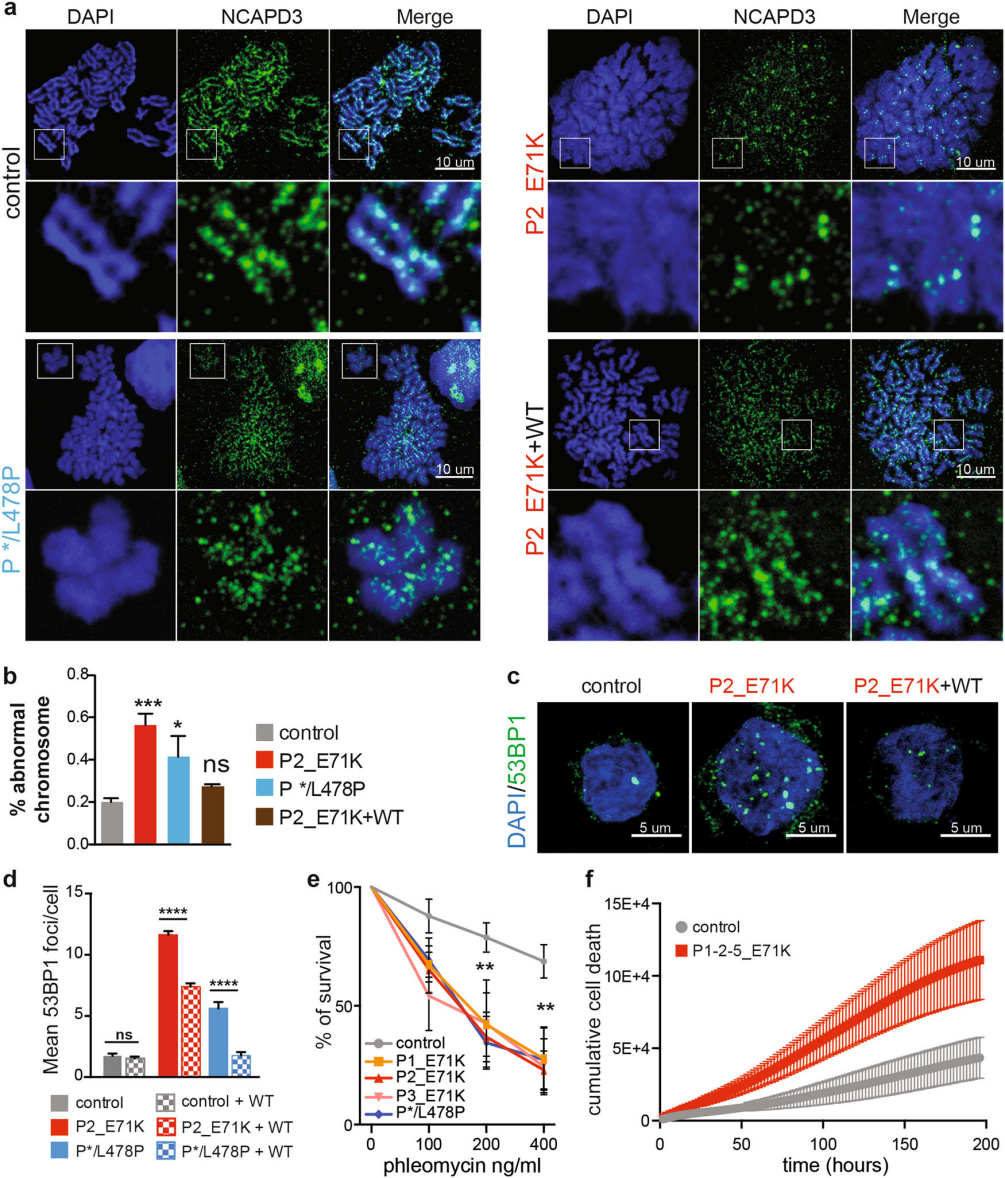
TTC7A deficiency affects genome integrity and survival. **a** Chromosome spreading of B lymphoblastoid cell lines (B-LCLs) blocked in metaphase using nocodazole. DNA is stained with DAPI and chromosome axis is delineated with NCAPD3 (a subunit of condensing II complex). Insets are depicted below each sample. Patient’s E71K B-LCLs re-expressing Flag-tagged WT_TTC7A is shown on the below right panel, scale bar: 10 µm. **b** Quantification of chromosomes with abnormal structure. **c** Assessment of 53BP1 foci formation in control and patient B lymphoblastoid cell lines (B-LCLs) non transduced or transduced with Flag-tagged WT_TTC7A. Left: Immunostaining of 53BP1 imaged by confocal microscopy, scale bar: 5 µm. **d** Quantification of 53BP1 foci number. Mean of three independent experiments  ± SEM. Total number of cells in control = 392; control + WT = 359; P2_E71K = 259; P2_E71K + WT = 241; P */L478 = 95; P */L478 + WT = 108; Tukey’s multiple comparisons test multiples ****p-value < 0.0001. Mean of four independent experiments ± SEM. Dunnett’s multiple comparisons test; ***p-value < 0.001; *p-value < 0.05. **e** Phleomycin sensitivity test. An increased dose of phleomycin was applied on B-LCLs, and survival was assessed 7 days later. Mean percentage  ± SEM; linear mixed-effects model fit by REML, *p* < 0.001. **f** Cumulative cell death upon CD4+ T-cell activation. Isolated naïve CD4+ T cells were activated with CD3/CD28. Cell growth and death was monitored using an IncuCyte ZOOM live cell imager (Essen Bioscience), starting at 24 h after activation and every 2 h during 9 days. Growth was measured by confluence phase-mask, and cytotoxicity by using Cytotox red fluorescence reagent. Two controls and three patients were analyzed; values are mean ± SEM. Tested using linear mixed-effects regression model, ANOVA, *P* < 0.0001418

We next evaluated the chromatin organization during mitosis, which represents the most condensed state of chromatin. At the onset of mitosis, the chromatin condenses and folds along a primary axis that contains key molecules like topoisomerase II and condensins^26^. Chromosome spreading studies and co-staining of the condensin II subunit NCAPD3 revealed a striking defect in chromosome condensation in P1–4-E71K_ and */L478P_TTC7A B-LCLs (Fig. 6a). The chromosomes had a swollen, fuzzy appearance. The NCAPD3 signal spanned the whole width of the chromatid and was no longer restricted to the central axis, as observed in WT_TC7A cells. In some cases, NCAPD3 signal along the chromosomal axis was lost (Fig. 6a). Despite this loss of chromatin condensation, sister chromatids and centromeres could still be distinguished in TTC7A mutated cells. Activated primary T lymphocytes from E71K_ and */L478P_TTC7A patients displayed the same anomalies (Supplementary Fig. S8a). Importantly, the induced expression of exogenous Flag-tagged WT_TTC7A in patient cells re-established chromosomal condensation and partially restored the NCAPD3 axis (Fig. 6b). These results indicate that TTC7A has a role in chromatin organization and/or condensation during both mitosis and interphase.

### TTC7A deficiency results in genome instability and impaired cell survival

A change in nucleosome organization can potentially affect genome stability because naked or non-nucleosomal DNA is more prone to induction of breaks and can also drive improper formation of abnormal chromatin structure that can elicit the DNA damage response. We thus assayed cells for DNA breaks in the interphase by visualizing foci of 53BP1, a master chromatin-associated repair factor. Immunostaining revealed the spontaneous formation of many multiple discrete 53BP1 foci in TTC7A mutant B-LCLs, primary T lymphocytes, and fibroblasts (Fig. 6c, Supplementary Fig. S8b, c). In contrast, 53BP1 foci did not accumulate in the patients’ cells following complementation with Flag-tagged WT_TTC7A (Fig. 6d), demonstrating that the increased susceptibility to DNA breaks observed in patient cells merely reflect an impairment in TTC7A function rather than a phenotype acquired by cell adaptation or selection. Consistently, TTC7A-deficient cells were highly sensitive to the radiomimetic drug phleomycin that is able to induce double-strand breaks in DNA (Fig. 6e). This was not a consequence of a major defect in the DNA repair machinery because the number of 53BP1 foci in patients’ cells returned to its initial level following irradiation, indicating the presence of delayed but effective repair (Supplementary Fig. S8d). Furthermore, we found that activated TTC7A-deficient CD4+ T cells were more prone to death than activated control cells (Fig. 6f). Altogether, these findings indicate that TTC7A deficiency increases the DNA’s accessibility and exposure to harmful endogenous or exogenous agents and thus has a negative impact on cell viability. This damage may account, at least in part, for the disease phenotype.

## Discussion

Based on various consistent experimental findings, our results reveal that TTC7A is an intrinsic component of the nucleus and an important regulator of chromatin structure and nuclear organization, with impacts on both transcriptional activity and genome stability. It remains to determine more precisely the molecular mechanisms by which TTC7A binds to chromatin and subsequently exerts its nuclear functions.

A large fraction of TTC7A is present in the nucleus, in both the nuclear matrix and chromatin-bound fractions. Several *TTC7A* mutations reported in severe human pathology result in depletion of the nuclear TTC7A pool in general and an absence of chromatin-bound TTC7A in particular. Importantly, complementation with wild-type TTC7A of patient cells harboring several distinct mutations rescued the defects of mutant cells, indicating a specific critical function of TTC7A that can be distinguished from an adaptation/selection of cells.

It is not known how TTC7A is targeted to or retained into the nucleus. Sequence analysis failed to identify a nuclear localization signal (NLS). However, other TPR-containing proteins (such as yeast SSN6, tetratricopeptide repeat domain 3 protein (TTC3), and TTC4) are known to localize to the nucleus. SSN6 is a repressor of gene expression in *Saccharomyces cerevisiae*^27^, TTC3 is an E3 ligase that binds to AKT and induces the latter’s proteasomal degradation within the nucleus^28^, and TTC4 is a putative tumor suppressor during melanocyte transformation and is involved in DNA replication^29,30^. Of these three proteins, only TTC3 contains a characterized NLS. Since all of the TTC7A natural mutants analyzed to date are excluded from the nucleus, we believe that these mutations directly impact nuclear import or force nuclear export. The C-terminal part of TTC7A is essential in this process, since TTC7A lacking the last 26 amino acids (P-A832*) is excluded from the nucleus. Strikingly, truncation of TTC7A—even when restricted to the last two amino acids—is associated with a severe form of the disease^31^. Hence, although the underlying mechanism remains to be characterized, TTC7A’s C-terminal part is critical for its biological function and is probably essential for its localization to the nucleus.

Our present results also show that TTC7A binds to chromatin within discrete genomic regions either indirectly through an as-yet-unknown molecular complex or directly through its TPRs. Indeed, proteins containing Huntington, EF3A, ATM, and TOR repeats (which are related to TPR domains) reportedly bind to the DNA directly, as is the case for condensins^32^. According to our results, TTC7A binding sites are enriched in highly transcribed areas of the genome encompassing most of differentially expressed genes found in TTC7A-deficient cells. Since TTC7A binds in intergenic sites of the genome, we hypothesize that TTC7A’s influence on transcription control is exerted at relative distance of genes that likely include enhancers.

Complex interactions between transcription factors, epigenetic modifiers, and chromatin structure regulate gene expression^33^. Here, we show that functional TTC7A is required for the development of an accurate epigenetic and transcriptional status at promoters and enhancers. In the absence of TTC7A, the observed transcriptional changes were correlated with histones PTM alterations in distal and proximal regulatory regions. It remains to be seen whether TTC7A (i) cooperates with histone-modifying enzymes to define epigenetic rearrangements or (ii) independently mediates epigenomic events.

Nevertheless, this type of change in transcriptional and epigenetic profile may impair various biological pathways in TTC7A-deficient cells. The analysis of the cells’ transcriptional patterns highlighted the deregulation of two main functions: inflammation/cell death and energy balance. We observed the upregulation of transcripts involved in the TNF signaling pathway, as well as in pro-apoptotic signaling that may contribute to the increased DNA damage seen in TTC7A-deficient cells. Variations in the expression of genes involved in glycolysis-regulated hypoxia and the mTORC1 signaling pathways were also observed. The latter are known to define the metabolic uptake, which switches according to the cell’s proliferation and differentiation states^34^. Likewise, these metabolic changes might underpin a previously described increase in cell differentiation in mini-gut organoids from patients^1^. Our findings pave the way for further investigations of the disease-causing molecular defects. One can hypothesize that TTC7A’s nuclear function is required in order to establish and/or safeguard cell identity during differentiation from a stem cell profile to a mature cell profile.

Chromatin organization in the nucleus proves to be involved in regulating genome function^35,36^, including cell fate^37–39^. We provide here the first evidence indicating that TTC7A is required for proper chromatin organization at the nucleosome level and for higher-order structures not only in the interphase nuclei, but also in mitotic chromosomes. Indeed, in absence of TTC7A, we detected an altered distribution of H2B, H3, and H4 between cytosolic and salt-extractable nuclear fractions and an increase of H1 amounts. This underlines clearly a defect in the histone management^40^. At the nucleosomal level, we detected alterations in the ratio chromatosome/core nucleosome accompanied by an increase of subnucleosomal DNA fragments, indicating that nucleosomal organization is more accessible in cells deficient for TTC7A. These observations are in line with a decrease of chromatin compaction both in the chromosomes and interphase cells nuclei.

Linker histones are known for their contribution to compaction of nucleosomal arrays^41,42^ and formation of higher chromatin structures such as the 30-nm fiber^43^. Our data indicating that chromatin appears less compacted in TTC7A when H1 level is increased is thus counterintuitive. In order to achieve compaction, H1 requires proper nucleosomal arrays organization. Since this was not observed in TTC7A-deficient cells, this function of H1 could not be fulfilled. Interestingly, H1 binding onto naked DNA is reported to cover a DNA length ranging from 10 to 65 bp^44–46^. An increase of the 55–75 bp fragments was detected in Mnase-digested DNA fragments from TTC7A-deficient cells. These fragments could represent DNA protected by transcription factors, but also H1-bound non-nucleosomal DNA. H1 is also involved in many nuclear processes including interplay with the HMG proteins, nucleosome remodeling activities, and higher-order chromatin domain organization (TADs)^20–22^. Our observations in a TTC7A-deficient context could thus reflect a change in the global organization of chromatin either as a specific mechanism promoted by TTC7A directly or also as perturbations of H1 functions, in response to increased H1 amounts. In such a scheme, H1 functions, not solely related to nucleosomal compaction, but involved in several nuclear processes, would lead to a change of chromatin organization and compaction.

Given that transcriptomic and proteomic experiments did not detect any alterations in known pathways involved in nucleosome dynamics, other as-yet-uncharacterized pathways are at work. Alternatively, TTC7A might exert a direct role at any of the steps involved in chromosomal compaction. Nucleosomes, topoisomerase II, and condensins are the main determinants of DNA supercoiling and chromosome compaction^47,48^. The potential involvement of TTC7A in these complexes warrants investigation. In addition, given the multifunctional aspects of TTC7A, we cannot totally rule out that the observed nuclear alterations are, at least in part, indirectly triggered by cytoplasmic events in which TTC7A might be involved as well. The alterations observed in TTC7A-deficient cells could result from the quantitative deficiency of TTC7A, but also from impaired function of the mutant residual protein or both. We also found that genome instability and spontaneous DNA breakage are accentuated in TTC7A-deficient cells. It is tempting to propose that this damage arises as a result of abnormal genome organization and greater exposure of naked DNA. These defects may decrease cell viability and thus account for some aspects of the pathology seen in TTC7A-deficient patients.

In conclusion, TTC7A appears to be an important chromatin-binding nuclear factor that contributes to normal chromatin compaction and proper nuclear organization. Its defect leads to unusual features reflecting an imbalanced distribution of core and linker histones. This function is particularly critical to ensure proper regulation of transcriptional program and genome stability with impact on cell fate determination and stability^37,38^. Although the molecular mechanisms through which TTC7A plays a role in this process has yet to be characterized, it is tempting to speculate that our results led to the identification of a novel factor modulating nucleosomal organization, with strong implication on human pathologies. Future work should seek to determine the detailed molecular mechanism through which TTC7A influences nuclear events and, ultimately, cell fate.

## Materials and methods

### Patients’ mutations and statements

Clinical information and blood samples were collected from the patients, their relatives, and controls, all of whom had given their prior informed consent to participation in the study. Genetic studies and data collection procedures were approved by the local investigational review board and the French Advisory Committee on Medical Research. Patients with E71K mutations were previously described and they belonged to a large consanguineous family (F1). Briefly, they carried a homozygous mutation on TTC7A gene; it consists of a hypomorphic missense with a substitution of the nucleotide 211 (G > A), leading to a change in the amino acid 71 from a glutamic acid to a lysine. The patient */L478P was also described and carried a deletion of the nucleotide 911 on one allele and a missense mutation with a substitution of the nucleotide 1433 (T > C) on the second allele. These changes lead to a frame shift and a substitution of amino acid 478 from a leucine to a proline (L478P), respectively. The patient */A524V carried newly found sporadic mutations that consist of a skip of exon 14 on one allele and a missense with a substitution of the nucleotide 1571 (C > T) on the second allele. These changes led to a loss-of-slice and a substitution of amino acid 524 from an alanine to a valine (A524V). The patient with A832* mutation was previously described and consists of a deletion at position 2496, leading to a premature stop codon at amino acid 832 that disrupts the last TPR domain of the protein. Patients annotations and correspondence with previously published annotations: P1_E71K = O3, P2 _E71K = K3, P3_E71K = F5, P4_E71K = D8, and P9_E71K = A3 belong to F1 family from Ref^6^. P5_E71K, P6_E71K, P7_E71K, and P8_E71K are unpublished new cases in F1. P */L478P = P3 are from F2 family from Ref^6^. P A832* = C3 and P ** = E3 belong to F and E families, respectively, from Ref^1^. P */A524V is an unpublished sporadic case.

### Patients-derived cells and culture conditions

Blood samples were collected from the patients and control subjects who had given prior informed consent to participation in the study. The local investigational review board and the French Advisory Committee on Data Processing in Medical Research approved the genetic studies and data collection procedures. Peripheral blood mononuclear cells (PBMCs) were isolated by density gradient centrifugation using Ficoll-Paque. B-lymphoblastoid cell lines (BLC) transformed with EBV, and primary fibroblast were prepared, as described previously *(1, 6)*. Naive CD4+ T cells were isolated from PBMCs using naive CD4+ T cell isolation kit II (Miltenyi Biotech 130–094–131), following the manufacturer's instructions. Primary T lymphocytes were stimulated for 2 days with phytohemagglutinin (PHA) (5 µg/ml; Sigma-Aldrich, St Louis, MO) or coated OKT3 with soluble CD28 antibody (1 µg/ml), and human interleukin-2 (IL-2) (50 to 200 IU/mL; PeproTech, Rocky Hill, NJ) in Panserin medium (PAN-Biotech GmbH, Aidenbach, Germany) supplemented with 10% human AB serum (S4190-100 Eurobio Life Science). Cells were cultured up to 6 or 7 days. B lymphoblast and fibroblast cell lines were cultured in RPMI medium 1640 + Glutamax (Gibco, ref 61870-010) supplemented with 10% (vol/vol) fetal bovine serum (Gibco), sodium pyruvate (1 mM, Life Technologies), and penicillin and streptomycin (100 U/ml each, Life Technologies).

### Antibodies

TTC7A primary antibody is a polyclonal antibody against human TTC7A prepared by immunizing rabbits with a TTC7A–GST fusion protein, and total immunoglobulin fraction was purified from serum (AgroBio, Saint-Aubin, France). Others antibodies used are: α-tubulin (Abcam, ab80779), p84 (Abcam, ab487), SP1 (Sigma, WH0006667M2), H2B (Abcam, ab1790), H3 (active motif, 39763), H4 (Abcam, ab31830), H1 (Abcam, ab61177), HA (Roche, 11 867 423 001), Flag (Sigma, F3165 and F1804), NCAPD3 (Novus, NB100-1573), 53BP1 (Novus, NB100-304), H3K27ac (active motif, 39685), H3K27me3 (active motif, 39155), and β-actin (Genetex, GTX629630). Secondary antibodies used are Horseradish peroxidase coupled to goat anti-rabbit, mouse or rat IgG (H + L), or Alexa Fluor 488, 555, 586, or 647 and purchased from molecular probes (Thermo Fisher).

### Constructs, electroporation, and transduction

The cDNA of E71K_TTC7A fused to 3*Flag and HA tag in the COOH terminus was generated from pcDNA-WT_TTC7A by using site-directed mutagenesis using QuikChange II XL Site-Directed Mutagenesis Kit (Agilent Technologies, 200521), following the manufacturer's instructions. B-lymphoblastoid cell lines (B-LCLs) were transfected using NEPA21 electroporator. Each electroporation was performed in 100-µl optiMem medium containing 2x10^6^ cells and 10 µg of plasmids.

For lentivirus production, WT_TTC7A-3*Flag-HA was subcloned into pWPI (addgene, #12254) vector as following: TTC7A-3*Flag-HA cDNA was amplified by PCR to add PmeI restriction site with the following primers: 5′-GTAAGCATGTTTAAACAAGCTTATGGCTGCGAAGGG-3′ and 5′-GTAAGCATGTTTAAACGGCGTAGTCTGGTACGTCGTATGGGTA-3′. PCR was performed by using the Taq Platinum (Invitrogen, 11304-011). Both TTC7A–PmeI PCR product and pWPI-ires-EGFP empty vector were digested with PmeI enzyme (Thermo Fisher, FD1344) and ligated by using T4 DNA Ligase (NEB, M0202M) following the manufacturer's instructions. The proper insertion of TTC7A-WT-3*Flag-HA into pWPI vector was verified by sequencing. pWPI-TTC7A-WT-3*Flag-HA-ires-EGFP vectors were used to produce TTC7A-WT-3*Flag-HA-ires-EGFP viral particles as following: HEK 293T cells were transfected with pWPI-TTC7A3*Flag-HA-ires-EGFP with the packaging plasmids phCMV-VSVG and pCMV-DR8.74 using lipofectamine 2000 (Thermo Fisher) according to the manufacturer’s instructions. pWPI, phCMV_VSVG and pCMV_delta_R8.74 vectors used in this production were a generous gift from Emmanuel Six (Marina Cavazzano Lab, Institute Imagine). Twenty-four hours after transfection, the first supernatant was discarded and fresh medium was added to the cells. The second day, supernatant was harvested, centrifuged at 2000 rpm for 10 min to discard the floating cells, and filtered through a 0.45-μm filter. Polybrene (Invitrogen, R21001) was added at a final concentration of 4 µg/ml. 2x10^6^ B lymphoblast cells were infected using 7 ml of the prepared supernatant and transferred to a well of six-well plate. Spinoculation (60 min centrifugation at 2000 rpm at RT) was performed. After 24 h, the supernatant was replaced with fresh medium. After 3 to 5 days, the cells were rinsed and sorted according to their GFP-positive signal. Equal number of negative and positive cells was collected. A second sorting was performed later in time to eliminate the remaining negative cells.

### Immunoprecipitation, mass spectrometry, and immunoblotting

For immunoprecipitation, cells were lysed in 50 mM HEPES, pH 8, 150 mM NaCl, 2 mM MgCl_2_, 1 mM EDTA, 0.5% Triton X-100, and 10% glycerol containing proteases and phosphatases inhibitors (Roche and Sigma). Collected cells were centrifuged for 15 min at 11,300 g. Supernatant was precleared with 50 µl of ½ slurry protein G-agarose beads (Thermo Fischer, 20398) coupled to normal rabbit IgG (Santa-Cruz, sc-2027) for 2 h at 4 °C. TTC7A antibody were applied to the precleared lysate with 50 µl of ½ slurry G-beads and incubated under rotation overnight. Resins were washed and proteins were eluted in 2 ×  loading reducing buffer (Thermo Fischer, 39000). Immunoprecipitations were analyzed on a SDS-PAGE, followed by immunoblotting and mass spectrometry sequencing. Equal amount of proteins were subjected to SDS-PAGE on polyacrylamide gels and transferred to PVDF membranes (Millipore, IPVH00010). Membranes were saturated with 5% BSA or 5% dry milk in TBS-0.1% Tween-20 for 30 min at RT. Primary antibodies were incubated for overnight at 4 °C and revealed with HRP-conjugated secondary antibodies, SuperSignal West Pico chemiluminescent substrate (Pierce), and exposure to film.

For immunoprecipitated and total protein levels analysis by mass spectrometry, proteins were digested overnight at 37 °C by sequencing grade trypsin (12.5 μg/ml; Promega Madison, WI, USA). Digests were analyzed by a LTQ Velos Orbitrap (Thermo Fisher Scientific, San Jose, CA) coupled to a nano-LC Proxeon 1000 system and an EASY-spray source (Thermo Fisher Scientific, San Jose, CA). Chromatographic separation of peptides was performed. Fragments were obtained with a collision-induced dissociation (CID). MS/MS spectra were acquired in the linear ion trap and were processed with Proteome Discoverer 1.4 software (Thermo Fisher Scientific, San Jose, CA) coupled to an in-house Mascot search server (Matrix Science, Boston, MA; version 2.4.2). Label-free analyses were performed with Progenesis-LC software 4.1 (non linear dynamics). WT and mutants conditions in triplicates were compared. After chromatogram alignments, peak detections and normalizations of the intensity of features, a step of identification by Mascot 2.4.2 (Matrix science, Boston, MA), were performed. Student’s t test was used to compare the protein levels between WT and mutant cells. Upregulated proteins were analyzed with Bingo and Reactome pathway tools from cytoscape software.

### Cellular protein fractionation

Cytoplasmic and nuclear extracts were prepared as following: cells were swollen in hypotonic buffer (10 mM HEPES.KOH [pH 7.5], 50 mM NaCl, 0.5 M sucrose, 0.1 mM EDTA, and protease inhibitors) for 20 min at 4 °C. An equal volume of hypotonic buffer containing 0.2% NP40 was added to the cell lysate, followed by quick 10 up and down. Lysate was centrifuged at 2000 rpm 5 min and the supernatant (cytoplasm) was recovered. The pelleted nuclei were washed three times (buffer composed of 10 mM HEPES pH 7.5, 10 mM KCl, 0.1 mM EDTA, 0.1 mM EGTA). Nuclei were lysed for 30 min with gentle mixing in buffer containing 10 mM HEPES pH 7.5, 500 mM NaCl, 0.1 mM EDTA, 0.1 mM EGTA, 0.1% NP40, and 500 U/ml benzonase enzyme. Total fractions were obtained after lysing cells for 15 min with 20 mM Tris-HCl, pH 8, 140 mM NaCl, 1 mM EDTA, 2 mM MgCl_2_, 1% NP40, and 500 U/ml benzonase enzymes. Centrifugation at 14,000 rpm for 15 min was performed on all fractions to discard the insoluble pellet. The subcellular protein fraction that enabled stepwise separation of cytoplasmic, membrane, nuclear, soluble, and chromatin-bound protein extracts were performed using the Thermo Fisher Kit (78840), following the manufacturer’s instructions.

### Imaging cell cytometer

Cells were electroporated as described above with pcDNA-TTC7A-3*Flag-HA_WT or E71K mutant. Two days post-transfection, cells were harvested and fixed in suspension with cold methanol for 10 min on ice. Cells were washed with PBS and post-fixed with 2% paraformaldehyde for 10 min at room temperature. Cells were pelleted at 3000 rpm for 5 min, washed twice in PBS, then blocked for 1 h with PBS + 0.1% tritonX100 + 5% FBS. After pelleting the cells, HA antibody was added overnight. After three washing steps, anti-rat-AF488 antibody was added for 1 h and DAPI for 5 min at room temperature. All cells were imaged using an Amnis ImageStream multispectral imaging flow cytometer system (Model IS100, Amnis). Following data acquisition, cells were gated and analyzed for TTC7A nuclear localization and nuclear area. Using the IDEAS® statistical analysis software, relative nuclear distribution of HA-TTC7A was assessed with the similarity score (Pearson’s correlation coefficient between the pixel intensities of the DAPI/HA image pair) and the area with the morphology erode mask.

### Microarray data analysis

Total RNA was freshly extracted from 2*10e6 cells using RNeasy mini-kit (QIAGEN). RNA quality was assessed with the Agilent High Sensitivity RNA ScreenTape System (Agilent Technologies): the RNA integrity number was > 9 for all the samples. Microarray experiments were performed on Affymetrix GeneChip® Human Transcriptome Array 2.0 following the manufacturer's instructions. Fluorescence data were imported into two analysis software: Affymetrix® Expression Console™ and R Bioconductor. Gene expression levels were calculated using the Robust Multiarray Averaging (RMA) algorithm of Expression Console, and flags were computed using a custom algorithm within R. All arrays in the dataset have good and reproducible quality metrics according to the standard guidelines. Assuming that a maximum of 80% of genes are expressed, we select the 20% lowest values for each microarray as background. A threshold is fixed at two standard deviations over the mean of the background. All probes, which normalized intensity measures were lower than the computed threshold, were flagged 0 instead of 1. The main list has been created, filtering probes flagged as “Present” (flag = 1) for at least half of the chips. Group comparisons were done using Student’s t test, and we filtered the resulting *p*-values at 5%. Heatmaps were generated using the R ctc package and Java TreeView software. Gene ontology analysis was performed using GSEA (http://software.broadinstitute.org/gsea/).

### Chromatin immunoprecipitation (ChIP)

For ChIP of histones modifications: 20e7 cells were double blocked in G1/S phase with thymidine double blockage (2 mM, 18-h first treatment, 9-h release, and 15-h second treatment). Cells were cross-linked for 10 min at RT with 1% formaldehyde. Quenching was performed with 125 mM glycine and cells were centrifuged at 2000 rpm for 10 min. Pellets were rinsed twice with PBS and lysed to isolate the nuclei. Nuclear extracts were sonicated to shear cross-linked DNA using the Bioruptor Pico Sonication System (Diagenode). Ten cycles of 30 s ON pulse 30 s OFF were sufficient to obtain DNA fragments around 150–200 bp in length. The resulting whole-cell extract was incubated for 2 h at 4 °C with 100-μl Protein G Agarose beads and 20 μg control IgG antibody to discard the unspecific binding. An aliquot corresponding to the input chromatin sample was isolated. Pre-cleared chromatin was immune-precipitated overnight with 20 μg antibodies against H3K27ac (39685, active motif), H3K27me3 (39155, active motif), and control IgG. Protein G Agarose beads, previously blocked with albumin and a mixture of DNA (polyA, polyC, yeast t-RNA, and salmon sperm DNA and *E. coli* DNA), were added for at least 3 h. Beads were washed five times with RIPA buffer and one time with TE containing 50 mM NaCl. Bound complexes and inputs were eluted from the beads in 100-μl TE + 0.1% SDS in a thermomixer for 1 h at 37 °C, twice. Cross-linking was reversed by incubating for 5 h at 65 °C. Immunoprecipitated DNA and whole-cell extract DNA were then purified by treatment with RNaseA, proteinase K, and Qiaquick PCR purification kit (28106).

For ChIP of Flag-TTC7A: two control B-LCLs were stably transduced with lentivirus expressing TTC7A_WT-3*Flag-HA-ires-EGFP as described above. ChIP was performed using two distinct Flag antibodies: IP1, sigma F1804 with 100*10e6 cells and IP2, sigma F3165 with 30*10e6 cells. ChIP was done similarly to above with the exception that cross-linking was performed with EGS (Thermo Fischer 21564) at 1.5 mM for 20 min then 10 min with 1% formaldehyde in PBS.

### Library preparation and sequencing

TruSeq ChIP kit from Illumina was used to perform library preparation according to the manufacturer’s recommendations. Briefly, IP and input DNA were first size selected using SPRIselect beads (Agencourt) in order to have fragments between 100 and 500 bp after BioAnalyzer profiling. one to ten nanogram of DNA were used in the first step of end-repair followed by the adenylation of 3′ end, allowing further ligation of specific Illumina adapters required for sequencing. These adapters contain specific indexes (barcodes) different for each sample, so that library from different samples can be mixed together before sequencing. After PCR amplification (12–15 cycles), quality assessment of DNA libraries was achieved using BioAnalyzer profiling and Qubit quantification. After equimolar pooling of libraries, the final dilution was accurately quantified using Kapa library quantification kit. Sequencing was performed on HiSeq 2500 instrument on rapid flow cells using single-read 50 or paired-read 100 nucleotides for reading mode SR50 or PE100 (see Table S1). Raw sequencing data were then used as Fastq files for further bioinformatic analysis. High-throughput sequencing was performed by the NGS platform of the Institute Curie supported by the grants ANR-10-EQPX-03, and ANR10-INBS-09-08 from the Agence Nationale de le Recherche and by the Canceropole Ile-de-France.

### ChIP-quantitative real time PCR (qRT-PCR)

PCR-based assays were performed to detect chromatin H3K27me3 and H3k27ac levels in patient cells stably expressing TTC7A_WT-3*Flag-HA-ires-EGFP. An abnormal methylated region in patient cells was analyzed using the following couple of primers: forward 5′-CTCGCCAAGTGCTCTCAGAA-3′ and reverse 3′-GGAAGGGACTCTTGCTTGCT-5′. An abnormally acetylated region in mutant cells was analyzed using the following couple of primers: forward 5′-AGCTGCTAGCCAGCAAATGT-3′ and reverse 5′-CTAATCCCCCTCAGGCAAGC-3′. qRT-PCR was performed using Fast SYBR green (Applied Biosystems) reagents. Serial dilutions of input DNA were used to perform standard curve amplification by qPCR in order to calculate a relative amount of enrichment. The fold enrichment was measured by dividing the ChIP signals by the IgG control-IP signals. Standard deviations were calculated from technical triplicate qPCR-reactions and represented as error bars.

### Bioinformatics analysis of NGS data

Sequencing data were processed in the main galaxy site (Galaxy Version 2.3.6.0) or with similar tools implemented in R packages. The quality of sequencing was checked using FastQC (http://www.bioinformatics.babraham.ac.uk/projects/fastqc/) and was close to 40. Raw reads were mapped against the human reference genome (hg38 or hg19 build) using Bowtie2 allowing up to 2 mismatches, and only alignments > = MQ30 and reads that are not marked as duplicates were considered. ChIP-seq peak calling was performed using MACS2, with–broad option for histone modification only and a q-value threshold of 0.05. Input data were used to model the background noise. Overlapped regions between the three controls and three patients were determined with BEDTools. The regions specifically methylated or acetylated in patients versus controls were delineated with BEDTools–v option. Reads coverage (bigwig) were normalized to 1 × (RPGC) according to effective genome size using bamCoverage tool. TTC7A binding sites and nearby genes were annotated with PAVIS program using default settings (https://manticore.niehs.nih.gov/pavis2/). Motif finding was performed with Homer R package (http://homer.ucsd.edu/homer/motif/). Ingenuity pathway analysis (IPA) (Ingenuity® Systems, http://www.ingenuity.com) was used to predict functional enrichment of genes (TSS) within 10 kb of TTC7A binding sites (peak summit). To visualize reads profile of H3k27ac or me3 nearby genes, we used ngs.plot package.

### Analysis of ChIP-seq combined to gene expression

We considered the start coordinate (TSS) of all the probe sets provided by HTA-2.0 array annotation (about 65000). TTC7A occupancy was performed in the vicinity of transcription start sites (TSS) of genes at promoters and at enhancers by using Meta-gene plot (R package). Identification of differential H3K27ac peaks between control and patients were determined with the ‘bdgdiff’ module of MACS2. Enhancers’ peaks were considered as H3K27ac peaks greater than 1 kb upstream and 10 kb downstream of TTS. Increased, decreased, and unchanged H3K27ac regions, respectively, were used to define the activated, repressed, and stable enhancers for subsequent analysis. Promoters’ peaks were considered, as H3K27ac peaks localized between 100 bp  upstream and 300 bp  dowstream TSS. H3K27ac occupancy at enhancers was performed by using meta-gene plot (R package).

The distribution of TTC7A binding sites on chromosomes compared with random chromosomal distribution was calculated by Cistrome Galaxy_CEAS tool.

### Immunocytochemistry

Cells were deposited on poly-lysine-coated coverslip and fixed with 2% paraformaldehyde in cold methanol for 2 min at −20 °C, then washed with PBS. After the blocking step (PBS, 0.1 % Triton X-100, and 5% FBS) of 2 h, primary antibodies were incubated in PBS, 0.1 % Triton X-100, and 1% BSA overnight at 4 °C. Coverslips were washed and secondary antibodies were incubated for 2 h at room temperature. Cells were counterstained with DAPI and mounted with proLong Gold anti-bleaching solution (Thermo Fisher, P36935). Dilutions of primary antibodies used are: HA 1/250, H3K27me3 1/100, and H3k27ac 1/50. For 53BP1 staining, cells were fixed in 4% PFA for 20 min, treated with 0.1 M glycine for 30 min, and primary antibody (1/100) were incubated for one an half hour at room temperature.

### Images acquisition and analysis

Images were acquired using a Leica SP8 or Zeiss LSM700 microscope with a HCL PL APO CS oil 63 x NA of 1.4 objective coupled to a piezzo and a coolSNAP HQ camera. Images were treated with ImageJ software. To measure DAPI staining variation within the nucleus, we employed an ImageJ macro that breaks down the image into equal ROI of 12 pixels each. The intensity of each ROI was quantified and the variations were calculated. Line Scan function of ImageJ was used to depict fluorescence intensity of H3K27me3 and H3K27ac along a line crossing the nucleus. Binary and mask function of ImageJ were used to determine the intensity of H3k27me3, first in the total nucleus, then in the central part in which delimitation was performed using a mask of the total eroded by 10 pixels. The border zone intensity was obtained by subtracting the central intensity from the total. For fluorescence lifetime imaging, we acquired photons de-excitation lifetime during 5 min. YoYo-1 (Thermo Fisher, Y3601, dilution 1/5000) displayed bi-exponential lifetime decay, and the fitting of the slope was around 1. The symphotime software measured the mean lifetime intensity in a region that delineated the nucleus. For 53BP1 foci formation, the focal plan  ± 2 stacks were Z-projected using ImageJ function, and 53BP1 foci were blindly counted.

### Chromosome spreading

Cells were blocked in metaphase using nocodazole at final concentration of 100 nM for 18 h. Cells were pelleted and suspended in a hypotonic solution containing 75 mM KCl for 12 min at 37 °C (water bath), then transferred on ice, and an equal volume of cold hypotonic solution containing 0.1% Tween20 was added in order to help spreading. Cells were immediately centrifuged in a cytofunnel chamber onto glass microscope slides for 5 min at 500 rpm. Slides were transferred to a Coplin jar containing KCM solution (120 mM KCl, 20 mM NaCl, 10 mM Tris HCl, pH 7.5, 0.1% TritonX100) freshly made and incubated for 7 min, then fixed for 10 min in 2% paraformaldehyde. Slides were washed with KCM solution and post-fixed with cold methanol 2 min at −20 °C. After PBS wash, slides were transferred for 1 h in a blocking solution (150 mM NaCl, 50 mM Tris-HCl, pH 7.5, 0.1% TritonX100, 5% fetal bovine serum). NCAPD3 antibody (1/50) was added on top of the slides and incubated overnight in a humid chamber at 4 °C. After three washes, anti-rabbit AF488 was added for 1 h at room temperature. Slides were mount with proLong gold anti-fade reagent and imaged.

### Histones extraction and quantification

Equal number of cells were swollen in hypotonic buffer (20 mM HEPES.KOH [pH 7.8], 5 mM potassium acetate, 0.5 mM MgCl_2_, and protease inhibitors) and disrupted with 25 strokes of a Dounce homogenizer. Following centrifugation (1 500 × g), the supernatant (cytosolic extract) was reserved, and the pelleted nuclei were incubated for 90 min with gentle mixing in hypotonic buffer containing 600 mM NaCl. Centrifugation at 20,000 × g for 20 min was performed to obtain a salt-extractable nuclear fraction and an insoluble pellet corresponding mainly to chromatin-bound histones. This latter pellet was lysed in SDS buffer containing benzonase enzyme to digest DNA and free histone-bound chromatin. Equal amounts of proteins were loaded on NuPAGE Electrophoresis System with Novex Bis-Tris precast 4–12% gradient gels and MES buffer (Invitrogen). The level of histones H2B, H3, H4, and the linker histone H1 were determined by western blot, and the quantification of chemiluminescence signal intensities were determined using the Gel-Pro analyzer. Intensities values were normalized to β-actin or α-tubulin. For each histone, control values were set to 1 and the relative histones’ levels in patients were calculated as compared with controls.

### Micrococcal nuclease digestion assay

Control and patients lymphocytes were harvested at 1x10^6^ cells per time point. Nuclei were isolated by hypotonic lysis, and micrococcal assay was performed using the episcope Nucleosome preparation kit (Takara, cat 5333) following the instructions of the manufacturer. DNA was extracted using nucleospin PCR clean-up kit (Macherey-Nagel, ref 740609). DNA size was assessed by using the Fragment Analyzer Automated CE System (Advanced Analytical). We used the High Sensitivity NGS Fragment Analysis Kit DNF-474 and PROSize® 2.0 Software to efficiently and accurately determine the size of the DNA and its concentration.

### Sensitivity test and cell survival

Cells were plated at 50,000 per well of p24 plate and cultured for 7 days upon exposure to increased doses of phleomycin (invivoGen, 11006-33-0). Survival was calculated by flow cytometer as the number of treated cells over untreated controls. For every treatment, data presented are the average of three independent experiments done in duplicate. For cumulative cell death, CD4+ were activated during 24 h, then transferred to poly-lysine-coated plate, and imaged for 9 days using the Incucyte Live-Cell Analysis System. IncuCyte® Cytotox Red Reagent (Cat No 4632, 1/5000) were added to label the dying cells in red. The number of red object per well was measured every 2 h, normalized to cell confluence measured by phase.

### Statistical analysis and data availability

Statistical analyses were conducted using Prism 6 software or R. Tests and *p*-values are indicated in figures legend. The primary microarray and NGS data sets have been deposited at the Gene Expression Omnibus (GEO) under accession numbers: E-MTAB-5934, E-MTAB-5940, and E-MTAB-5938.

## Electronic supplementary material

Supplementary Information

Supplementary Table S1

## Electronic supplementary material

**Supplementary Information** accompanies the paper at (10.1038/s41421-018-0061-y).

## References

[1] Bigorgne AE (2014). TTC7A mutations disrupt intestinal epithelial apicobasal polarity. J. Clin. Invest..

[2] Agarwal NS (2014). Tetratricopeptide repeat domain 7A (TTC7A) mutation in a newborn with multiple intestinal atresia and combined immunodeficiency. J. Clin. Immunol..

[3] Avitzur Y (2014). Mutations in tetratricopeptide repeat domain 7A result in a severe form of very early onset inflammatory bowel disease. Gastroenterology.

[4] Chen R (2013). Whole-exome sequencing identifies tetratricopeptide repeat domain 7A (TTC7A) mutations for combined immunodeficiency with intestinal atresias. J. Allergy Clin. Immunol..

[5] Fernandez I (2014). Multiple intestinal atresia with combined immune deficiency related to TTC7A defect is a multiorgan pathology: study of a French-Canadian-based cohort. Medicine.

[6] Lemoine R (2014). Immune deficiency-related enteropathy-lymphocytopenia-alopecia syndrome results from tetratricopeptide repeat domain 7A deficiency. J. Allergy Clin. Immunol..

[7] Samuels ME (2013). Exome sequencing identifies mutations in the gene TTC7A in French-Canadian cases with hereditary multiple intestinal atresia. J. Med. Genet..

[8] Woutsas S (2015). Hypomorphic mutation in TTC7A causes combined immunodeficiency with mild structural intestinal defects. Blood.

[9] Spiering D, Hodgson L (2011). Dynamics of the Rho-family small GTPases in actin regulation and motility. Cell Adhes. & Migr..

[10] Baird D, Stefan C, Audhya A, Weys S, Emr SD (2008). Assembly of the PtdIns 4-kinase Stt4 complex at the plasma membrane requires Ypp1 and Efr3. J. Cell. Biol..

[11] Scheufler C (2000). Structure of TPR domain-peptide complexes: critical elements in the assembly of the Hsp70-Hsp90 multichaperone machine. Cell.

[12] D’Andrea LD, Regan L (2003). TPR proteins: the versatile helix. Trends Biochem. Sci..

[13] Baskin JM (2016). The leukodystrophy protein FAM126A (hyccin) regulates PtdIns(4)P synthesis at the plasma membrane. Nat. Cell Biol..

[14] Fessing MY (2014). Gene regulation at a distance: higher-order chromatin folding and the coordinated control of gene transcription at the epidermal differentiation complex locus. J. Invest. Dermatol..

[15] Horn PJ, Peterson CL (2002). Molecular biology. Chromatin higher order folding–wrapping up transcription. Science.

[16] Maison C, Quivy JP, Probst AV, Almouzni G (2010). Heterochromatin at mouse pericentromeres: a model for de novo heterochromatin formation and duplication during replication. Cold Spring Harb. Symp. Quant. Biol..

[17] Rawlings JS, Gatzka M, Thomas PG, Ihle JN (2011). Chromatin condensation via the condensin II complex is required for peripheral T-cell quiescence. EMBO J..

[18] Estandarte AK, Botchway S, Lynch C, Yusuf M, Robinson I (2016). The use of DAPI fluorescence lifetime imaging for investigating chromatin condensation in human chromosomes. Sci. Rep..

[19] Mazumder A, Roopa T, Basu A, Mahadevan L, Shivashankar GV (2008). Dynamics of chromatin decondensation reveals the structural integrity of a mechanically prestressed nucleus. Biophys. J..

[20] Hergeth SP, Schneider R (2015). The H1 linker histones: multifunctional proteins beyond the nucleosomal core particle. EMBO Rep..

[21] Crane-Robinson C (2016). Linker histones: History and current perspectives. Biochim. Biophys. Acta.

[22] Fyodorov DV, Zhou BR, Skoultchi AI, Bai Y (2018). Emerging roles of linker histones in regulating chromatin structure and function. Nat. Rev. Mol. Cell Biol..

[23] Cole HA (2016). Novel nucleosomal particles containing core histones and linker DNA but no histone H1. Nucleic Acids Res..

[24] Henikoff JG, Belsky JA, Krassovsky K, MacAlpine DM, Henikoff S (2011). Epigenome characterization at single base-pair resolution. Proc. Natl. Acad. Sci. USA.

[25] Kent NA, Adams S, Moorhouse A, Paszkiewicz K (2011). Chromatin particle spectrum analysis: a method for comparative chromatin structure analysis using paired-end mode next-generation DNA sequencing. Nucleic Acids Res..

[26] Ono T (2003). Differential contributions of condensin I and condensin II to mitotic chromosome architecture in vertebrate cells. Cell.

[27] Schultz J, Marshall-Carlson L, Carlson M (1990). The N-terminal TPR region is the functional domain of SSN6, a nuclear phosphoprotein of Saccharomyces cerevisiae. Mol. Cell. Biol..

[28] Suizu F (2009). The E3 ligase TTC3 facilitates ubiquitination and degradation of phosphorylated Akt. Dev. Cell..

[29] Dmitriev RI, Okkelman IA, Abdulin RA, Shakhparonov MI, Pestov NB (2009). Nuclear transport of protein TTC4 depends on the cell cycle. Cell Tissue Res..

[30] Crevel G, Bennett D, Cotterill S (2008). The human TPR protein TTC4 is a putative Hsp90 co-chaperone which interacts with CDC6 and shows alterations in transformed cells. PLoS. One..

[31] Yang W (2015). Compound heterozygous mutations in TTC7A cause familial multiple intestinal atresias and severe combined immunodeficiency. Clin. Genet..

[32] Piazza I (2014). Association of condensin with chromosomes depends on DNA binding by its HEAT-repeat subunits. Nat. Struct. Mol. Biol..

[33] Bonev B, Cavalli G (2016). Organization and function of the 3D genome. Nat. Rev. Genet..

[34] Pollizzi KN (2016). Asymmetric inheritance of mTORC1 kinase activity during division dictates CD8(+) T cell differentiation. Nat. Immunol..

[35] Gibcus JH, Dekker J (2013). The hierarchy of the 3D genome. Mol. Cell.

[36] Hubner MR, Eckersley-Maslin MA, Spector DL (2013). Chromatin organization and transcriptional regulation. Curr. Opin. Genet. & Dev..

[37] Becker JS, Nicetto D, Zaret KS (2016). H3K9me3-Dependent Heterochromatin: Barrier to Cell Fate Changes. Trends Genet.: TIG.

[38] Gonzalez-Sandoval A, Gasser SM (2016). On TADs and LADs: Spatial Control Over Gene Expression. Trends Genet.: TIG.

[39] Cheloufi S (2015). The histone chaperone CAF-1 safeguards somatic cell identity. Nature.

[40] Gurard-Levin ZA, Quivy JP, Almouzni G (2014). Histone chaperones: assisting histone traffic and nucleosome dynamics. Annu. Rev. Biochem..

[41] Stockley PG, Thomas JO (1979). A nucleosome-like particle containing an octamer of the arginine-rich histones H3 and H4. FEBS Lett..

[42] Fan Y (2005). Histone H1 depletion in mammals alters global chromatin structure but causes specific changes in gene regulation. Cell.

[43] Finch JT, Klug A (1976). Solenoidal model for superstructure in chromatin. Proc. Natl. Acad. Sci. USA.

[44] Yue H, Fang H, Wei S, Hayes JJ, Lee TH (2016). Single-molecule studies of the linker histone H1 binding to DNA and the nucleosome. Biochemistry.

[45] Mamoon NM, Song Y, Wellman SE (2002). Histoneh1(0) and its carboxyl-terminal domain bind in the major groove of DNA. Biochemistry.

[46] Watanabe F (1986). Cooperative interaction of histone H1 with DNA. Nucleic Acids Res..

[47] Baxter J (2011). Positive supercoiling of mitotic DNA drives decatenation by topoisomerase II in eukaryotes. Science.

[48] Charbin A, Bouchoux C, Uhlmann F (2014). Condensin aids sister chromatid decatenation by topoisomerase II. Nucleic Acids Res..

