# Supplementary material for: Tetratricopeptide repeat domain 7A is a nuclear factor that modulates transcription and chromatin structure
Source: Cell Discov. 2018 Nov 13;4:61. doi: 10.1038/s41421-018-0061-y (PMC6232119; doi:10.1038/s41421-018-0061-y)
Supplement: Supplementary file 1 — Supplementary Information [file 41421_2018_61_MOESM1_ESM.pdf]

Supplementary Information

Supplementary Figures and Figure legends

Supplementary Figure 1

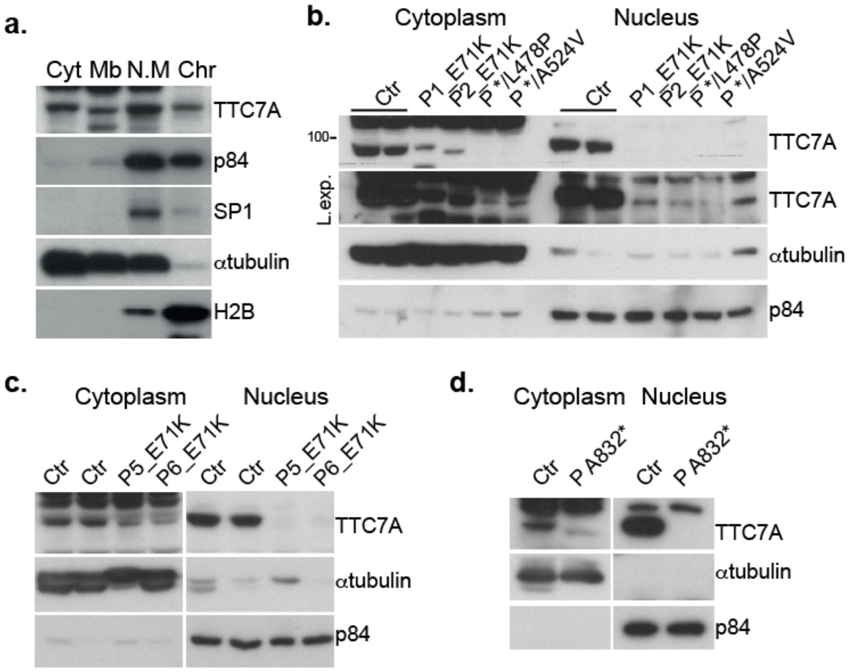

Supplementary Figure 1. Nuclear localization of TTC7A is highly decreased in mutant cells.

(**a**) Isolated subcellular fractionation were subjected to western blot and assessed for TTC7A level and specific antibodies for each compartment. The nucleoplasm protein p84, the transcription factor SP1, α-tubulin and histone H2B were used to monitor the purity of isolated fractions. Cytoplasm (Cyt), membrane (Mb), nuclear matrix (N.M), chromatin-bound (chr). (**b**) Cellular fractionation of B lymphoblastoid cell lines from 2 healthy donors (ctr) and 4 patients (P1-2_E71K; P */L478P and P */A524V). TTC7A (96 kDa) expression level was assessed in cytoplasmic and nuclear fractions by western blot. Long exposure (L.exp.) for TTC7A blot is also shown. α-tubulin and the nucleoplasm protein p84 were used to monitor the purity of isolated fraction. (**c**, **d**) Cellular fractionation of primary T lymphoblast activated in culture. Peripheral blood mononuclear cells from healthy donors and patients (P5_E71K, P6_E71K and P A832*) were isolated and stimulated for 7 days in culture. Cytoplasm and nuclear fractions were subjected to western blotting and assessed for TTC7A level and specific antibodies for each compartment.

Supplementary Figure 2

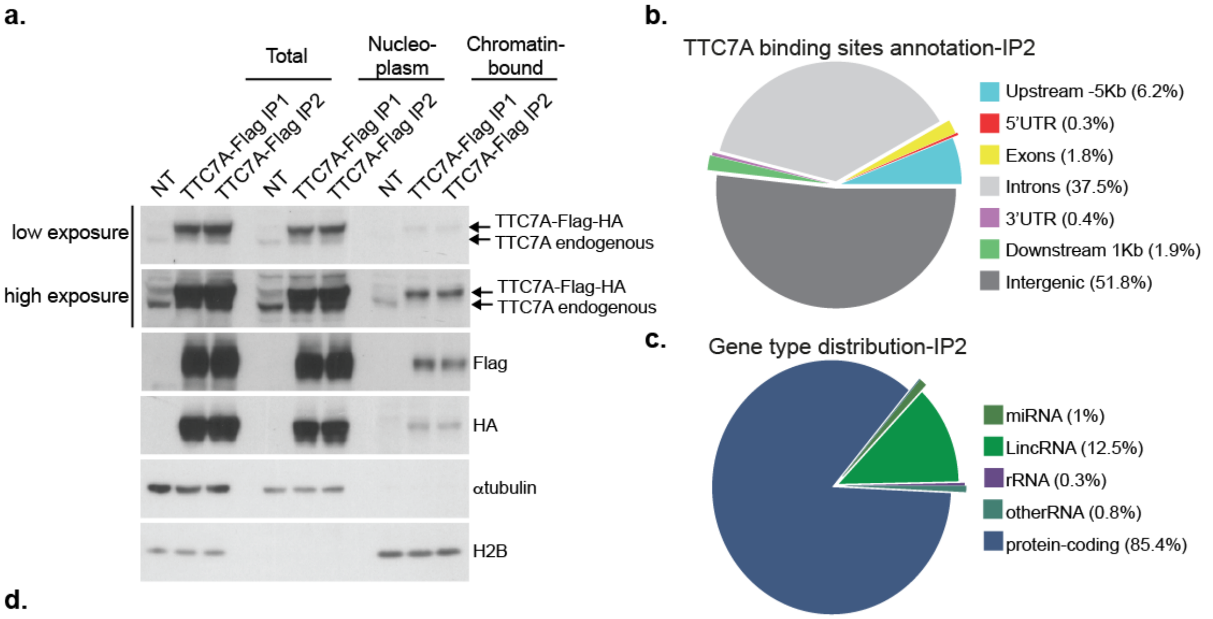

d.

TTC7A binding sites Motif Finding_IP1/2

Rank	Motif	P-value	% of Targets	% of Background	STD(Bg STD)	Best Match/Details
1	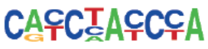	1e-671	55.00%	2.44%	54.1bp (172.6bp)	Egr1(Zf)/K562-Egr1-ChIP-Seq(GSE32465)/Homer(0.666)
2	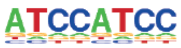	1e-664	56.42%	2.77%	56.0bp (178.1bp)	HOXA2(Homeobox)/mES-Hoxa2-ChIP-Seq(Donaldson_et_al.)/Homer(0.757)

Supplementary Figure 2. Genomic characterization of TTC7A binding sites.

(**a**) Level of exogenous expression of WT_TTC7A-Flag-HA constructs *vs* endogenous TTC7A in control B lymphoblastoid cell lines stably transduced. TTC7A protein level was assessed by western blot in total, cytoplasmic and nuclear fractions. (**b**) ChIP-seq peaks annotation of TTC7A IP2. (**c**) Gene type distribution of nearby genes for TTC7A peaks from IP2. TTC7A enriched peaks are associated with the genes whose TSS is the closest and genes features category is provided. (**d**) Binding motif analysis of TTC7A genomic sites (common peaks of IP1 and IP2) by using HOMER de novo motif discovery tool.

Supplementary Figure 3

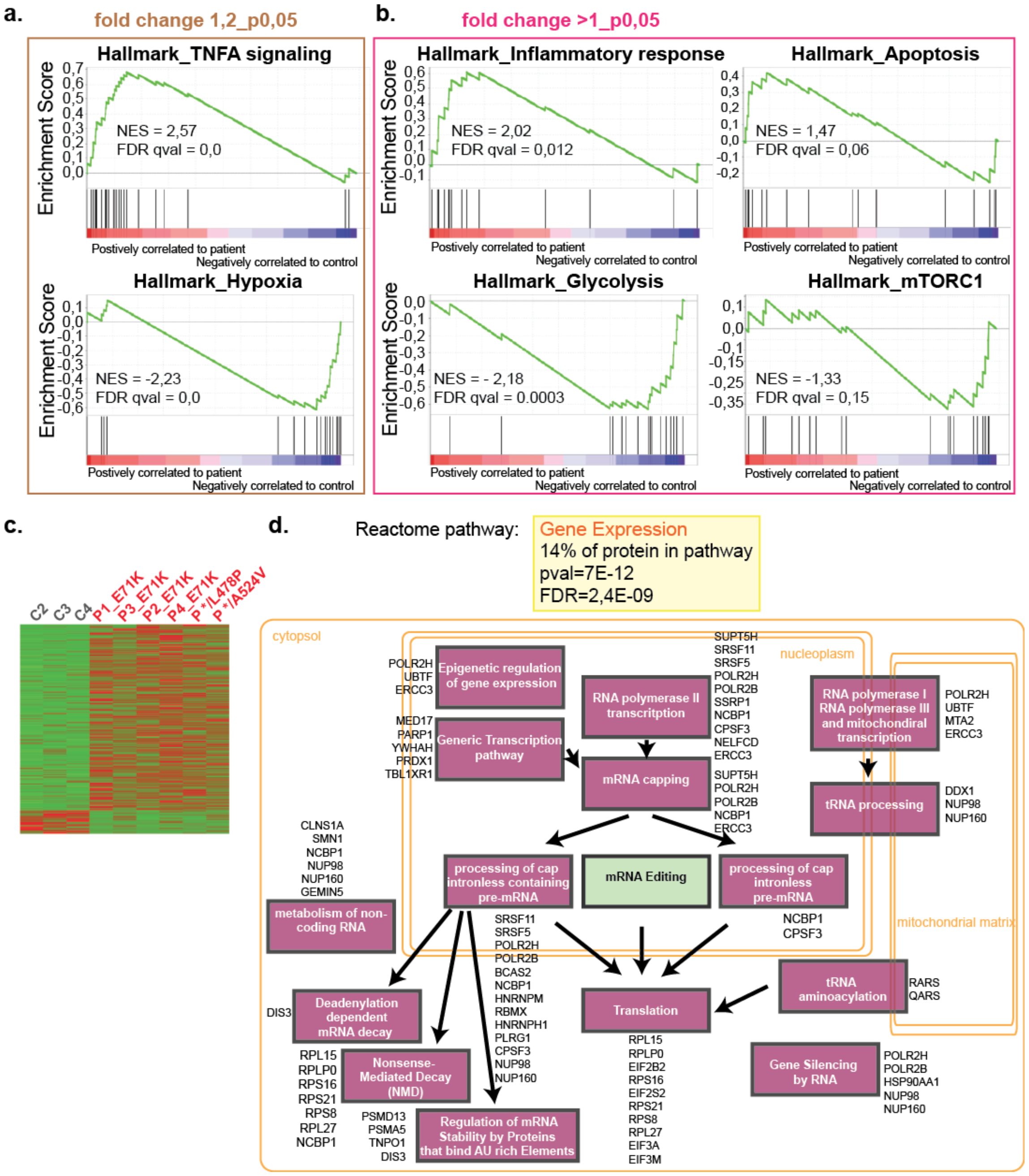

Supplementary Figure 3. Functional enrichment of differentially expressed genes in TTC7A-deficient cells.

(**a**) Gene set enrichment analysis (GSEA)-enrichment plots of representative gene sets from molecular signature database Hallmark of TNF signaling and Hypoxia. The list of differentially expressed genes with a fold change of 1.2 and a p-value of 0.05 is considered. NES: Normalized enrichment score; FDR: false discovery rate. (**b**) GSEA analysis is performed on the list of differentially expressed genes with a p-value of 0.05, same as (A). (**c**) Heat map illustrates differentially abundant proteins (p<0.05; student t test) determinate by

mass spectrometry (green: low; red: high). Proteins from total cellular lysates of controls (C2 to C4) and 6 patients (P1 to 4_E71K, P*/L478P and P*/A524V) were quantified and normalized by label free. (**d**) Reactome pathway depicting the set of proteins up-regulated in patient. The gene expression pathway was the highest functional enrichment.

Supplementary Figure 4

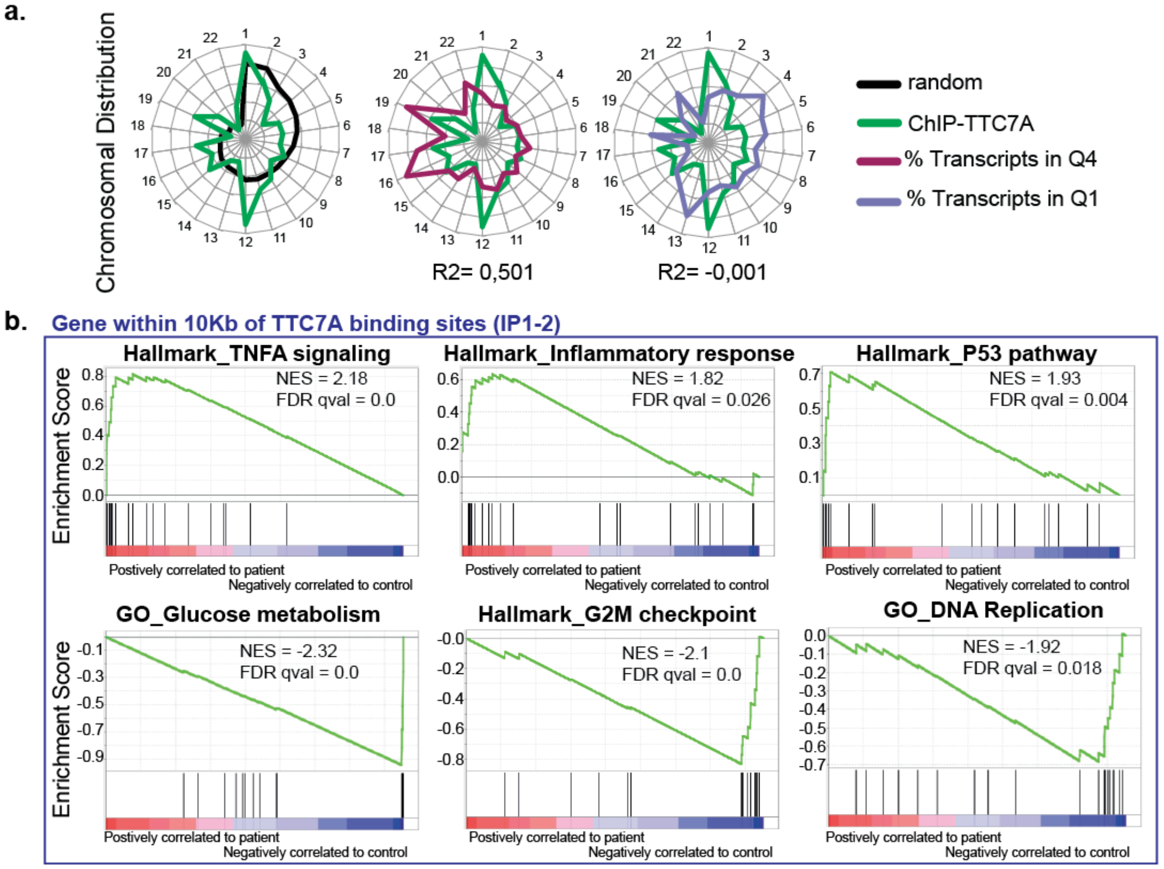

Supplementary Figure 4. Non-random genomics distribution of TTC7A binding sites and transcriptional deregulation of nearby genes.

(**a**) Chromosomal distribution of TTC7A binding sites as compared to (left) a random distribution across the 22 chromosomes of the genome, middle: transcripts belonging to the 4th quartile of expression level (Q4), right: transcript belonging to the 1th quartile of expression level (Q1). R-squared indicate the statistical measure of how close the data are to the fitted regression lines. (**b**) Gene set enrichment analysis (GSEA)-enrichment plots of representative gene sets from molecular signature database Hallmark or Gene Ontology (GO). The list of genes localized with 10 kb upstream and downstream of TTC7A peaks' center is considered.

Supplementary Figure 5

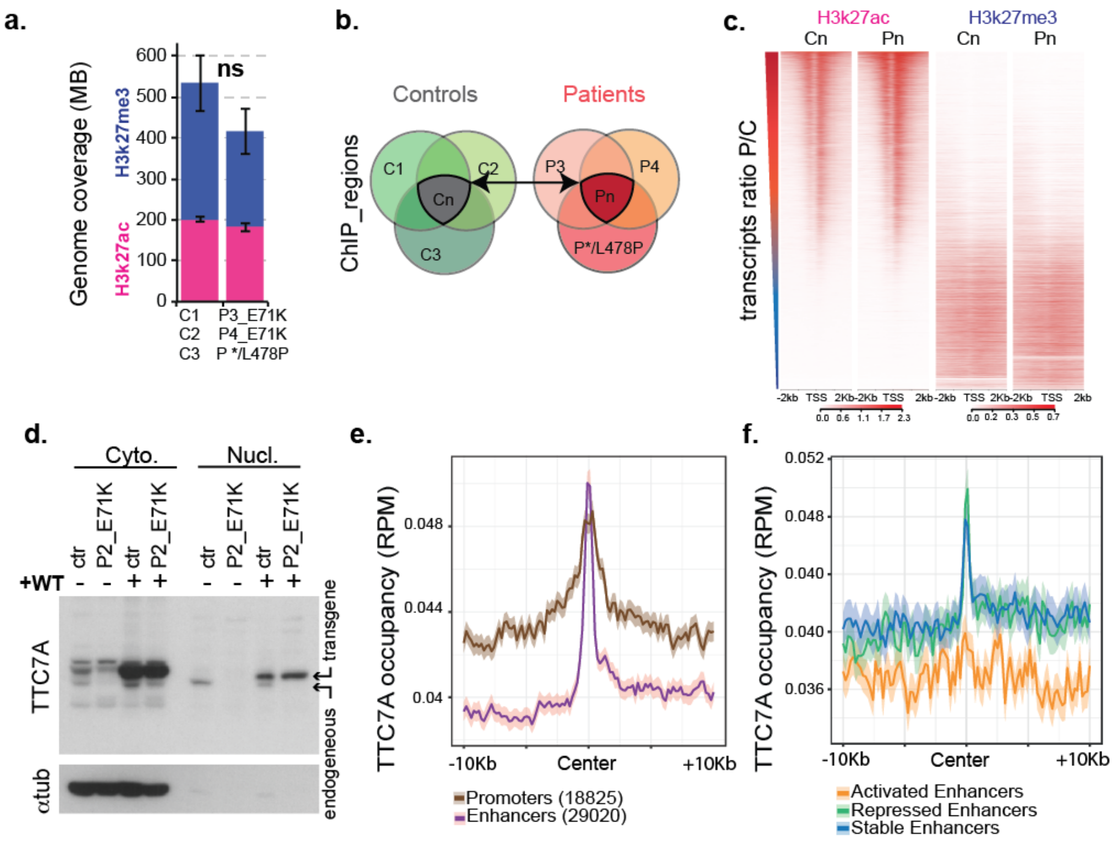

Supplementary Figure 5. H3K27 acetylation, tri-methylation and TTC7A binding analysis at transcriptional regulatory elements.

(**a**) Genomic coverage of H3k27ac (pink) and H3k27me3 (blue) regions in control (C1 to C3) and patients (P3_E71K, P4_E71K and P*/L478P). Mean +/- SEM, ns: non-significant. MB stands for megabase. (**b**) Genomic regions marked by H3k27ac and H3k27me3 and shared between controls (Cn) on one hand, and patients (Pn) on other hand, were determined using intersectbed tool. (**c**) Density heat maps for H3k27ac and H3k27me3 reads count +/- 2 kb of TSS. Color intensity represents normalized and globally scaled tag counts. TSSs are ranked according to patient over control gene expression ratio (P/C). Maps emphasize the mutually exclusive trend of both markers. (**d**) Level of exogenous expression of WT_TTC7A Flag tagged constructs in control and patients' B lymphoblastoid cell lines stably transduced. TTC7A protein level was assessed by western blot after cytoplasmic and nuclear fractionation. (**e**) Metaplots of TTC7A occupancy at promoters and enhancers determined in control cells based on the H3K27ac enrichment and localization. Intergenic H3K27ac peaks (greater than 2 kb upstream of TSS and 10 kb downstream of TTS) were considered as enhancers. The promoter is defined as the H3K27ac region covering 100 bp upstream to 300

bp downstream of the TSS. RPM: reads per million mapped reads. (**f**) Metaplots of TTC7A occupancy at activated, repressed and stable enhancers determined in control cells.

Supplementary Figure 6

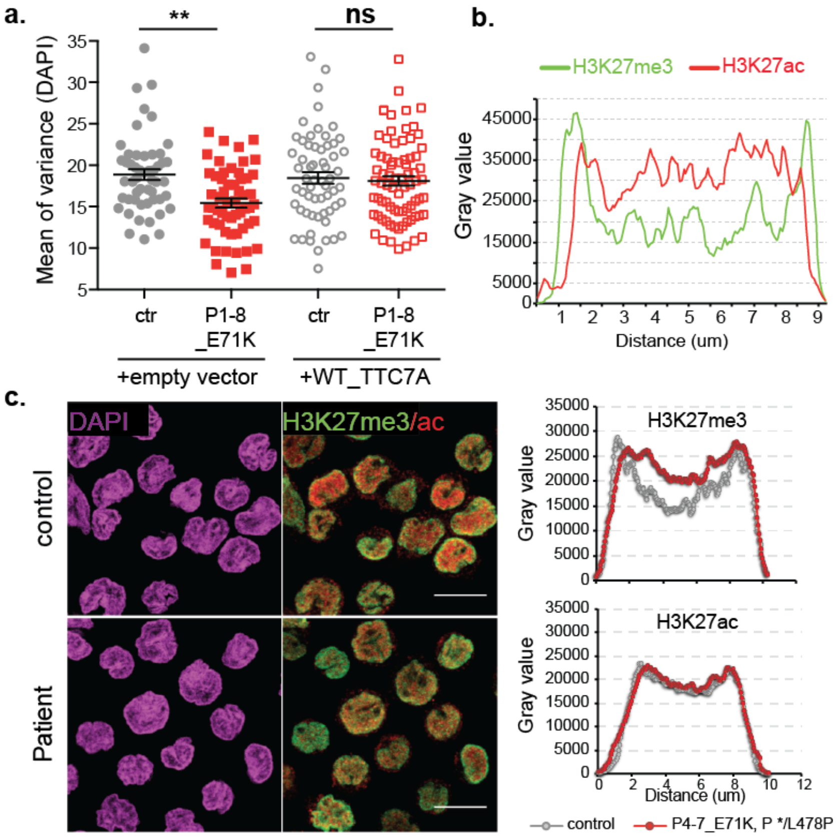

Supplementary Figure 6. Nuclear repartition of H3K27 tri-methylation is altered in mutant TTC7A.

(**a**) DAPI intensity variation in control and patient CD4 T cells. Cells were isolated from peripheral blood of two controls and two TTC7A-deficient patients, and transduced with particles coding either for the GFP alone or WT_TTC7A-ires-GFP. Upon activation, cells were analyzed for chromatin compaction at 7 days of culture. DAPI nuclear mask were applied and each nucleus was sub-divided into squares of 12 pixels size in order to calculate DAPI intensity variance. Mean of two controls and 2 patients +/-SEM. Total number of activated cells from controls + empty vector (n=51) and patients + empty vector (n=60), and from controls + WT_TTC7A (n=56) and patients + WT_TTC7A (n=77). Ordinary one way ANOVA multiple comparisons p-value<0.01. (**b**) Intensity plots profile of H3k27me3 and H3K27ac along a line crossing the nucleus of representative control lymphocyte. (**c**) Immunostainings of histone 3 lysine 27 tri-methylation (H3k27me3, green) and acetylation (H3K27ac, red) of activated T lymphocytes. Graphs show mean intensity plots profile of H3k27me3 or acetylation along a line crossing the nucleus (mean of 20 cells per samples).

Supplementary Figure 7

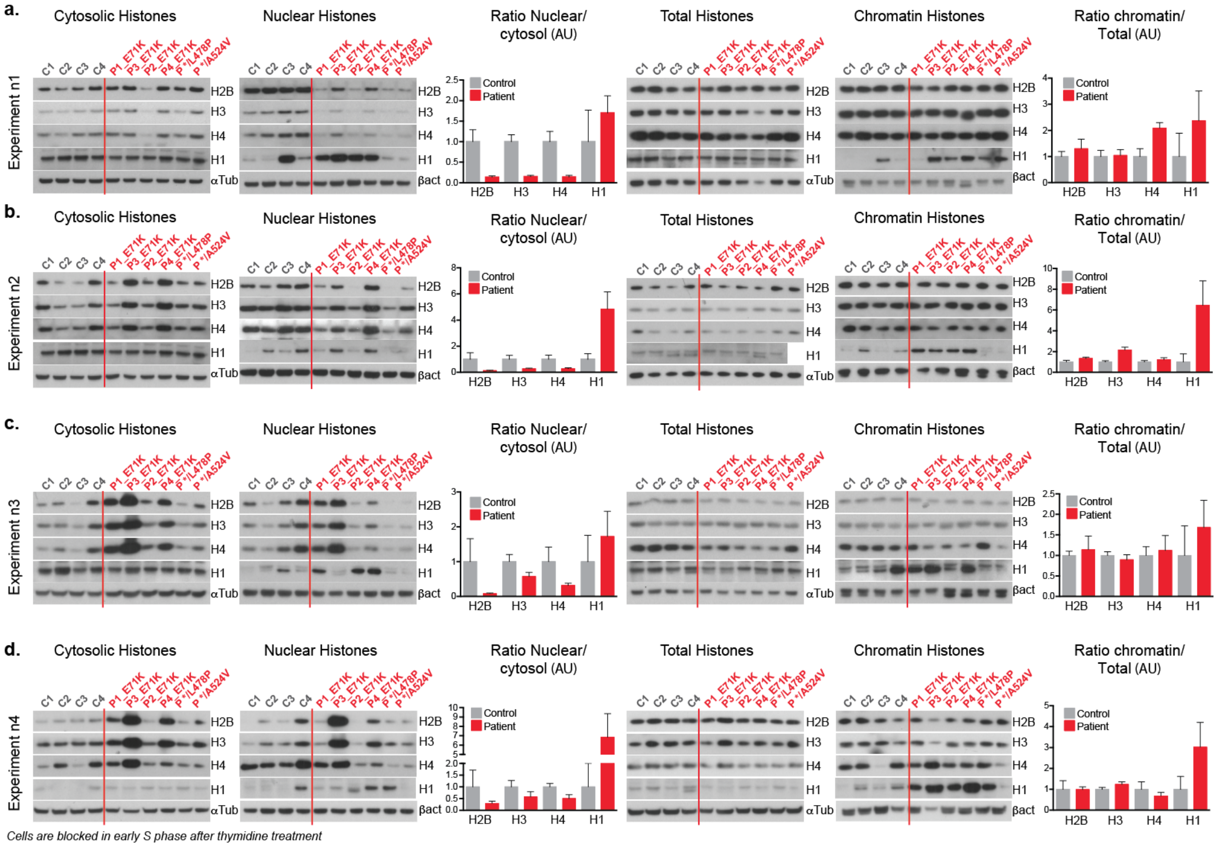

Supplementary Figure 7. TTC7A is required for the proper histones distribution.

(**a-d**) Histones subcellular fractionation in control and patient B lymphoblastoid cell lines. Histones H2B, H3, H4 and H1 were analyzed by western blot. Fractions are total histones, cytosolic histones (soluble pool), nuclear histones (salt-extractable pool), and chromatin histones (DNA enwrapped pool). A to C are experiments performed on routine cell in culture, while experiment D is performed on cells synchronized in early S phase upon thymidine treatment. The 4 controls are compared to the 6 patient samples and quantification is relative to control that is set to 1. The signal intensities of histones was normalized to internal loading control, αtubulin (αtub) for total and cytosolic fractions; βactin (βact) for nuclear and chromatin fractions. Graphs represent the ratio between nuclear *vs* cytosolic, and chromatin-bound *vs* total pools.

Supplementary Figure 8

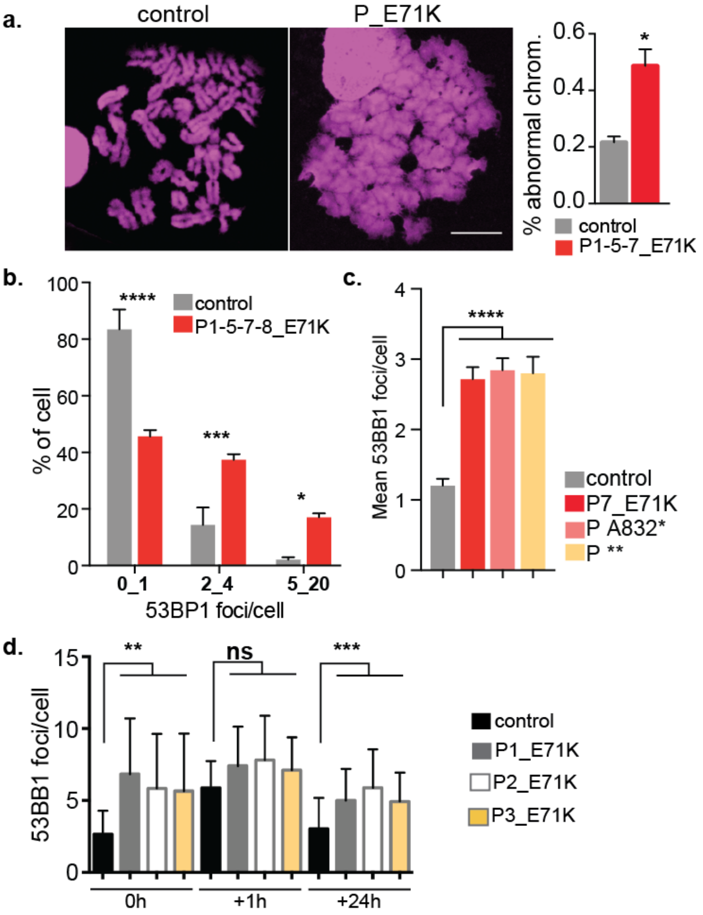

Supplementary Figure 8. Genomic instability in TTC7A-deficient cells.

(**a**) Chromosome spreading and DAPI staining of activated T lymphocytes blocked in metaphase with nocodazole. Right: quantification of mitotic abnormal chromosomes. Mean of two independent experiments +/-SEM. Unpaired t test; * p-value=0.0407. (**b**, **c**) 53BP1 foci count in primary activated T lymphocytes (b), and primary fibroblasts (c). For b, mean of four independent experiments +/-SEM. Sidak's multiple comparisons test; **** p-value=0.0001, *** p-value=0.001; * p-value=0.05. For c, mean of two independent experiments +/-SEM. Dunnett's multiple comparisons test, ** p-value<0.01. (**d**) Kinetics of irradiation-induced 53BP1 foci formation in B lymphoblastoid cell lines from controls and patients (irradiation=0,5 Gy). Mean +/-SEM. Total number of cells >30; t test; *** p-value<0.001; * p-value<0.01.
